# Supplementary material for: Minimal vertical transport of microplastics in soil over two years with little impact of plastics on soil macropore networks
Source: Commun Earth Environ. 2025 Apr 9;6(1):278. doi: 10.1038/s43247-025-02237-w (PMC11981934; doi:10.1038/s43247-025-02237-w)
Supplement: Supplementary file 2 — Supplemental material for publication (PDF file) [file 43247_2025_2237_MOESM2_ESM.pdf]

Supporting Information

Minimal vertical transport of microplastics in soil over two years with little impact of plastics on soil macropore networks

Roman B. Schefer[1], John Koestel[2], Denise M. Mitrano[1*]

[1] Environmental Systems Science Department, ETH Zürich, Universitätstrasse 16, 8092 Zürich, Switzerland

[2] Soil Quality and Soil Use, Agroscope, Reckenholzstrasse 191, 8046 Zürich, Switzerland

Corresponding author: denise.mitrano@usys.ethz.ch

Summary:

Fifteen pages

Twelve figures (S1 – S12)

Twenty-five tables (S1 – S12.3)

Further information is provided on: Temporal evolution of soil morphologies in ROI 3 (Figure S1), impact of MPs on soil morphologies in ROI 1 (Figure S2 and Figure S3), impact of MPs on soil morphologies in ROI 2 (Figure S4 and Figure S5), impact of MPs on soil morphologies in ROI 3 (Figure S6 and Figure S7), temperature and precipitation data (Figure S8), random column distribution in the field (Figure S9), visual insights of the field site (Figure S10), X-ray scanner setup and reconstruction parameters (Table S1), histogram of calibrated grey values from X-ray images of different years (Figure S11), impact of MPs on crop yield (Figure S12), statistical analysis of soil morphologies (Table S2.1 – Table S10.2), statistical analysis of vertical transport of MPs (Table S11.1 - Table S11.3), statistical analysis of effect of MPs on crop yield (Table S12.1 - Table S12.3).

S1. Temporal evolution of soil morphologies in ROI 3

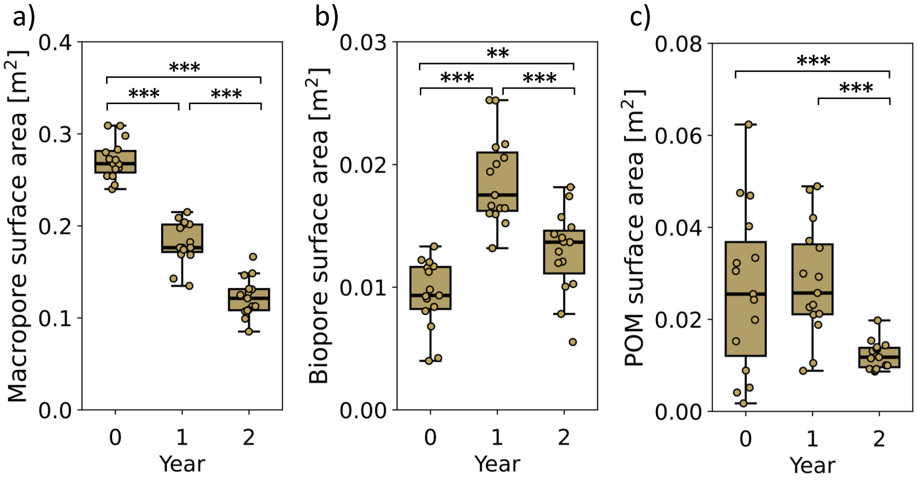

Figure S1: Temporal evolution of the macropore surface area (a), biopores surface area (b) and POM surface area (c). Trends observed in the surface areas for the different morphologies are consistent with those previously noted in the volume fractions. Boxplots show dots: individual data points, line: median, box: lower and upper quartile, whisker: highest and lowest value, empty dots: outlier. Statistical significance is indicated as follows: p-value <0.001 ***, <0.01 **, and <0.05 *.

S2. Impact of MPs on evolution of soil morphologies in ROI 1

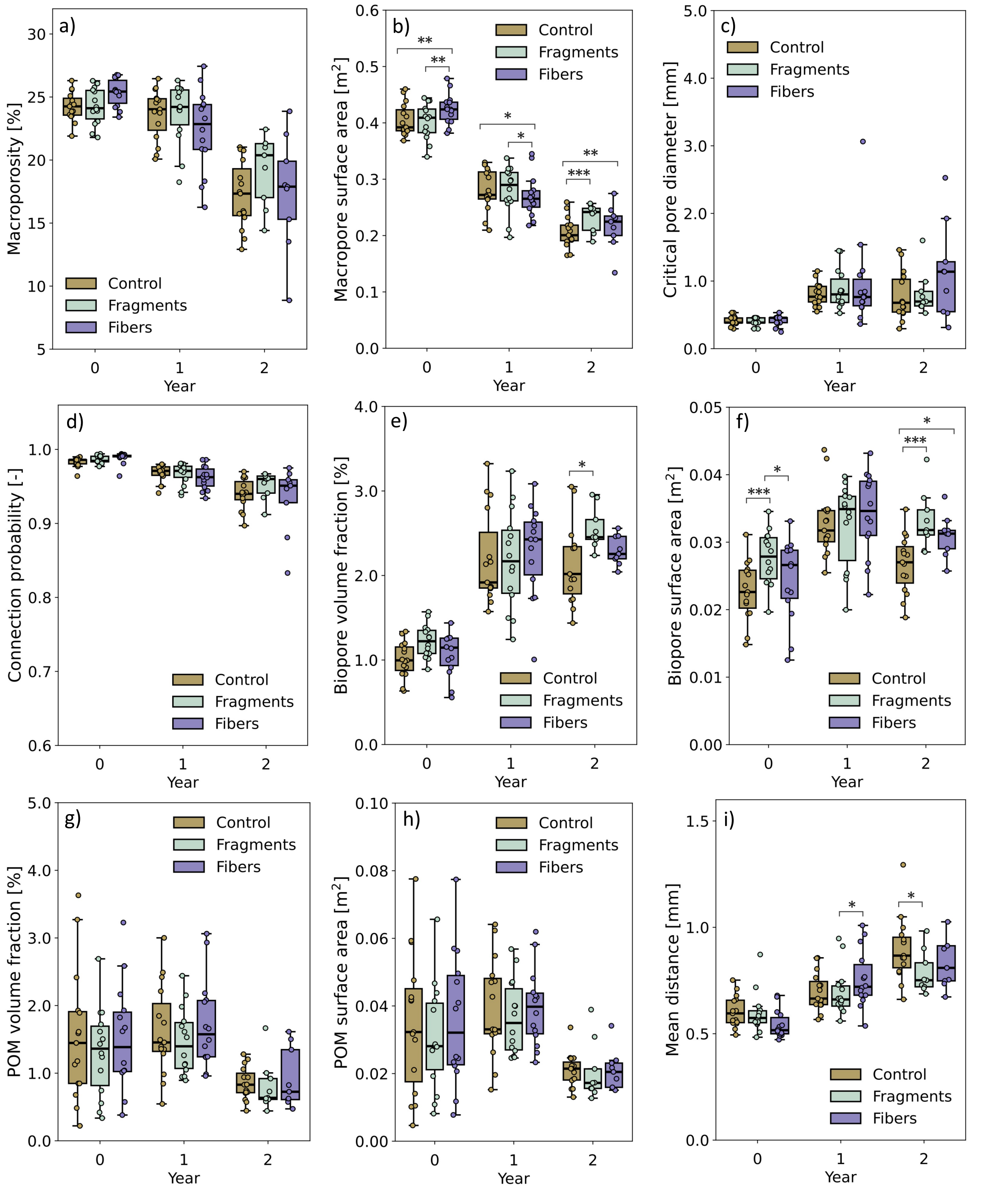

Figure S2: Evolution of soil morphologies in ROI 1 over two years for control (beige), fragment (cyan) and fiber (violet). Panels showing: Macroporosity (a), macropore surface area (b), critical pore diameter of macropores (c), connection probability of macropores (d), biopore volume fraction (e), biopore surface area (f), POM volume fraction (g), POM surface area (h) and mean distance of soil matrix to next aerated macropore connected to the top (i). Dots: individual data points, line: median, box: lower and upper quartile, whisker: highest and lowest value, dots outside whisker: outlier. Statistical significance is indicated as follows: p-value <0.001 ***, <0.01 **, and <0.05 *.

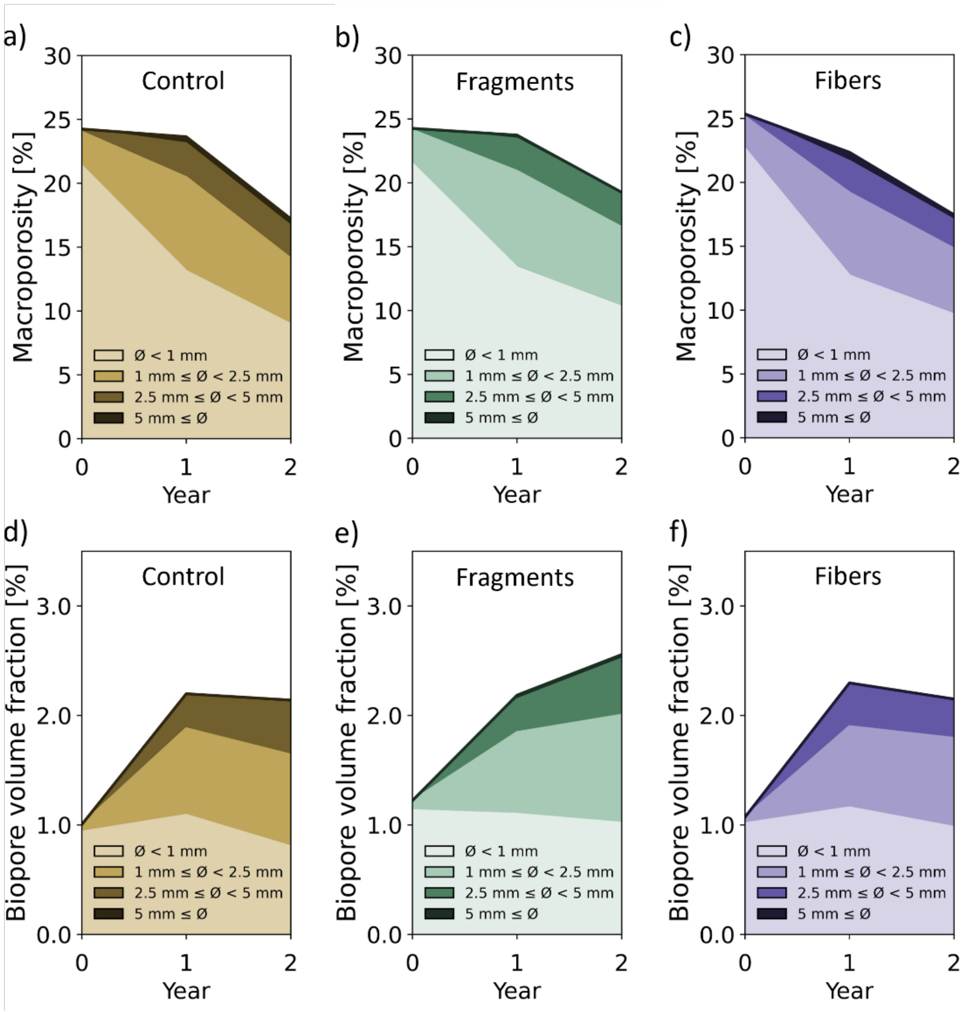

Figure S3: Evolution of size distribution of macropores (panels a-c) and biopores (panel d-f) in ROI 1 over two years.

S3. Impact of MPs on evolution of soil morphologies in ROI 2

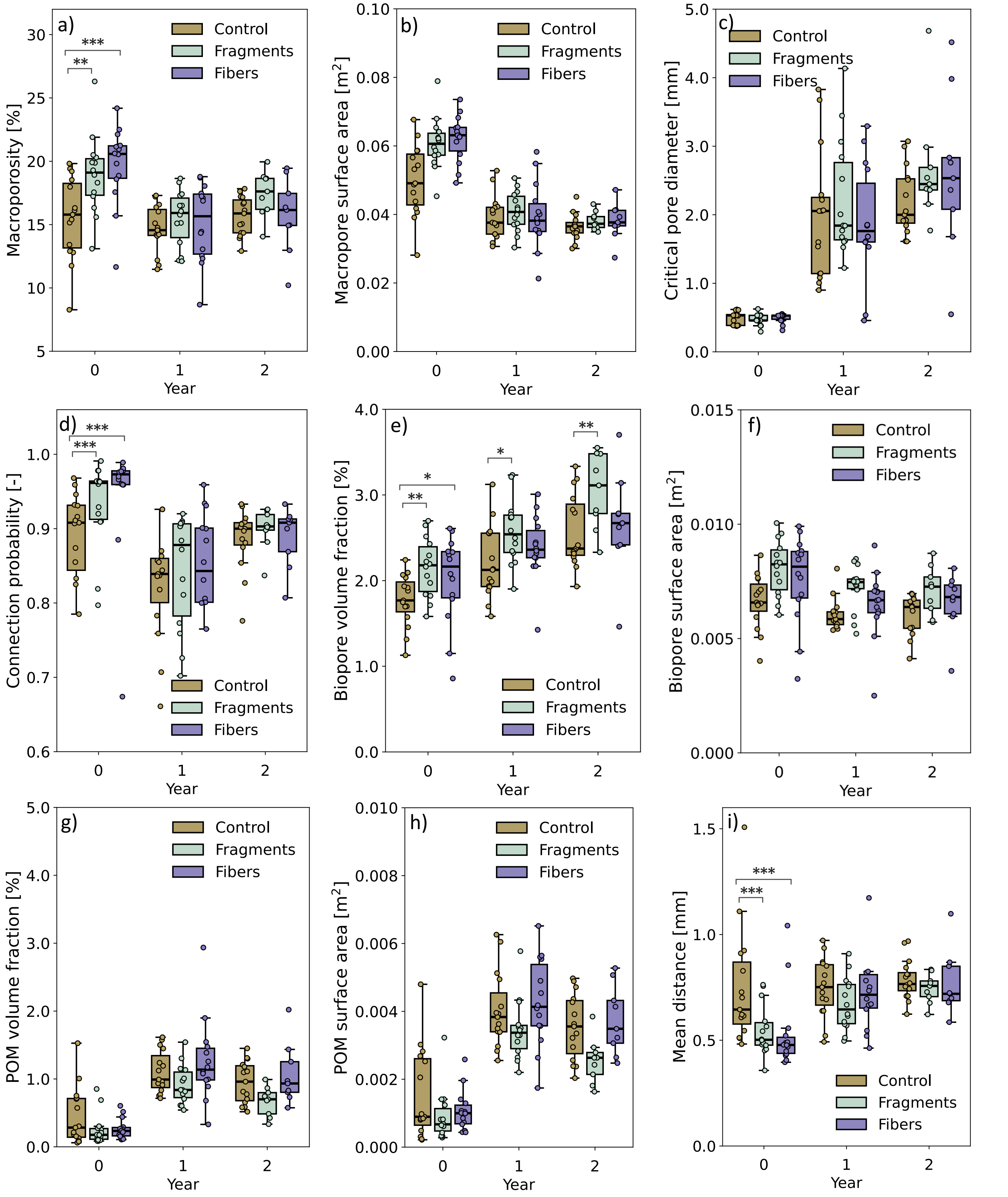

Figure S4: Evolution of soil morphologies in ROI 2 over two years for control (beige), fragment (cyan) and fiber (violet). Panels showing: Macroporosity (a), macropore surface area (b), critical pore diameter of macropores (c), connection probability of macropores (d), biopore volume fraction (e), biopore surface area (f), POM volume fraction (g), POM surface area (h) and mean distance of soil matrix to next aerated macropore connected to the top (i). Dots: individual data points, line: median, box: lower and upper quartile, whisker: highest and lowest value, dots outside whisker: outlier. Statistical significance is indicated as follows: p-value <0.001 ***, <0.01 **, and <0.05 *.

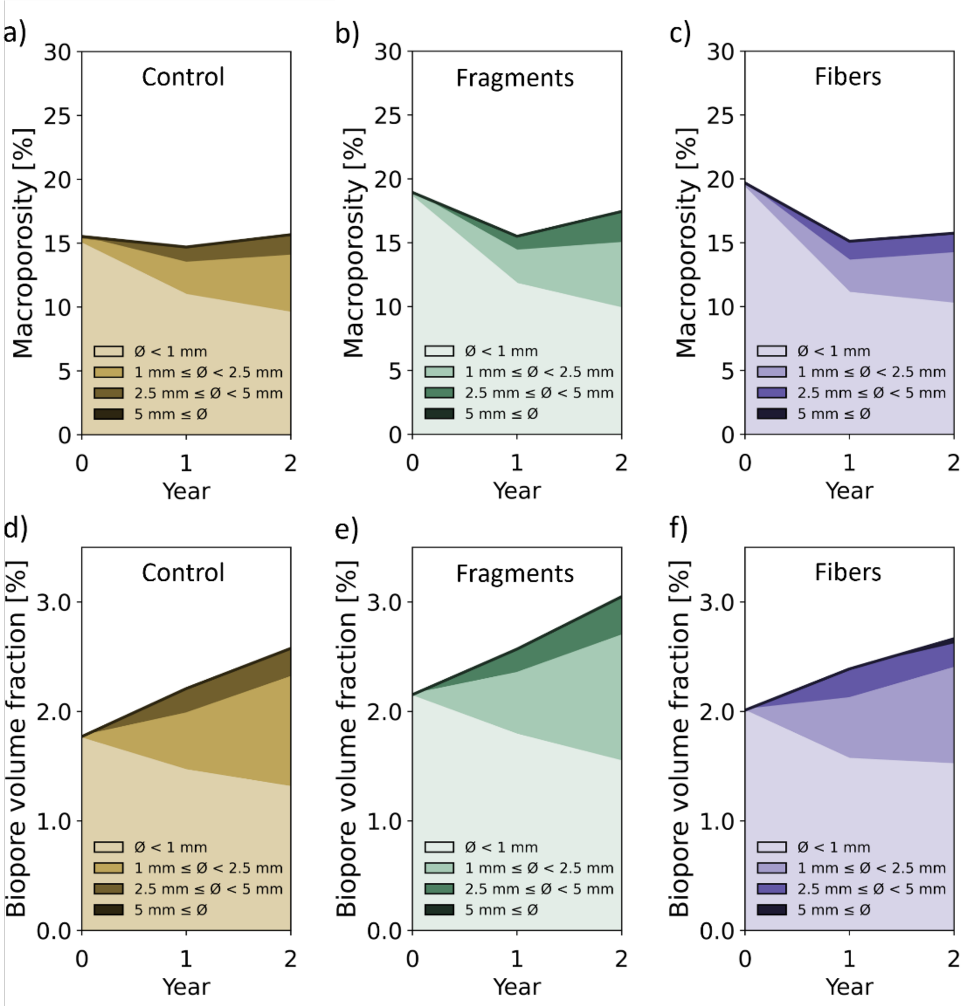

Figure S5: Evolution of size distribution of macropores (panels a-c) and biopores (panel d-f) in ROI 2 over two years.

S4. Impact of MPs on evolution of soil morphologies in ROI 3

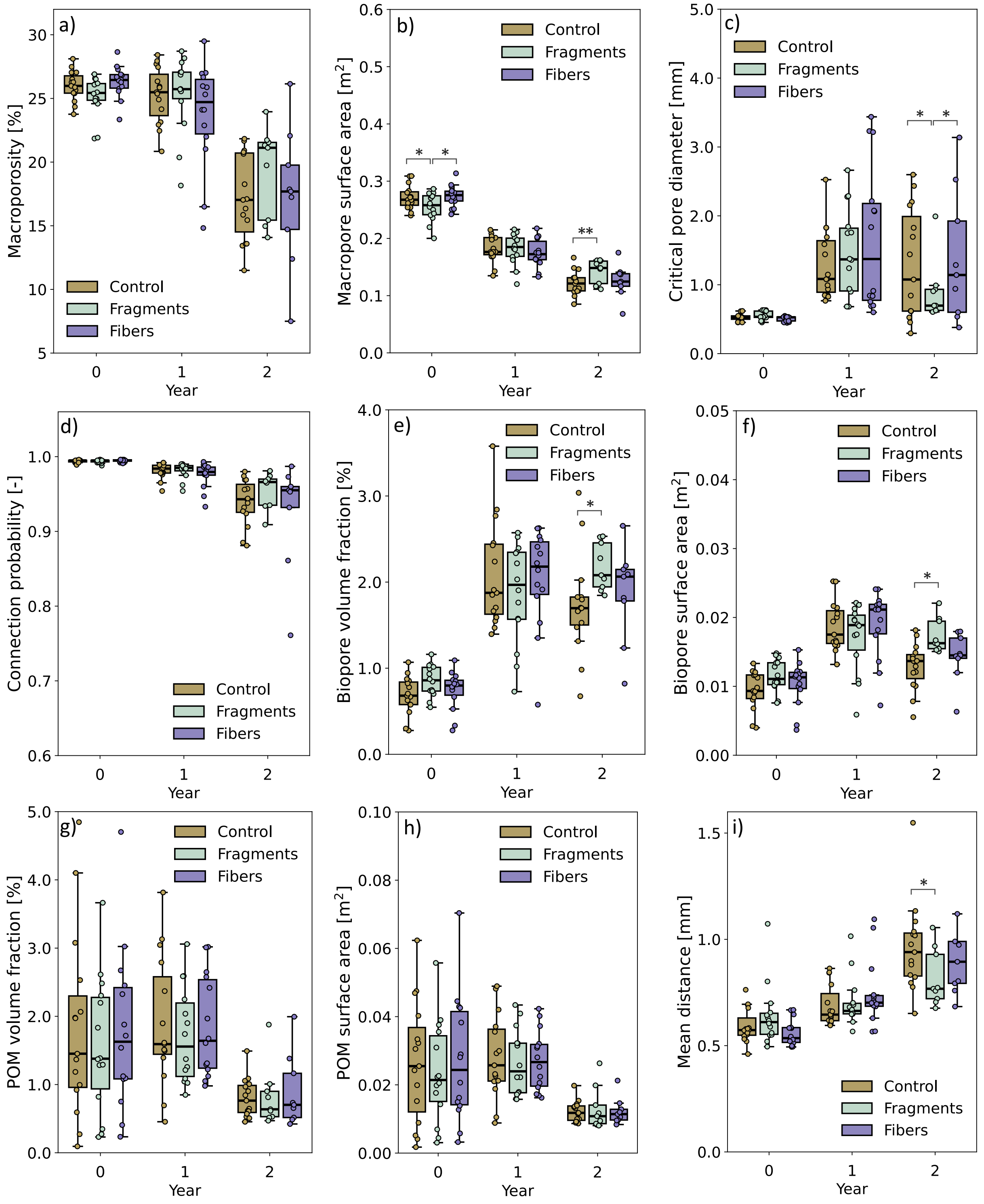

Figure S6: Evolution of soil morphologies in ROI 3 over two years for control (beige), fragment (cyan) and fiber (violet). Panels showing: Macroporosity (a), macropore surface area (b), critical pore diameter of macropores (c), connection probability of macropores (d), biopore volume fraction (e), biopore surface area (f), POM volume fraction (g), POM surface area (h) and mean distance of soil matrix to next aerated macropore connected to the top (i). Dots: individual data points, line: median, box: lower and upper quartile, whisker: highest and lowest value, dots outside whisker: outlier. Statistical significance is indicated as follows: p-value <0.001 ***, <0.01 **, and <0.05 *.

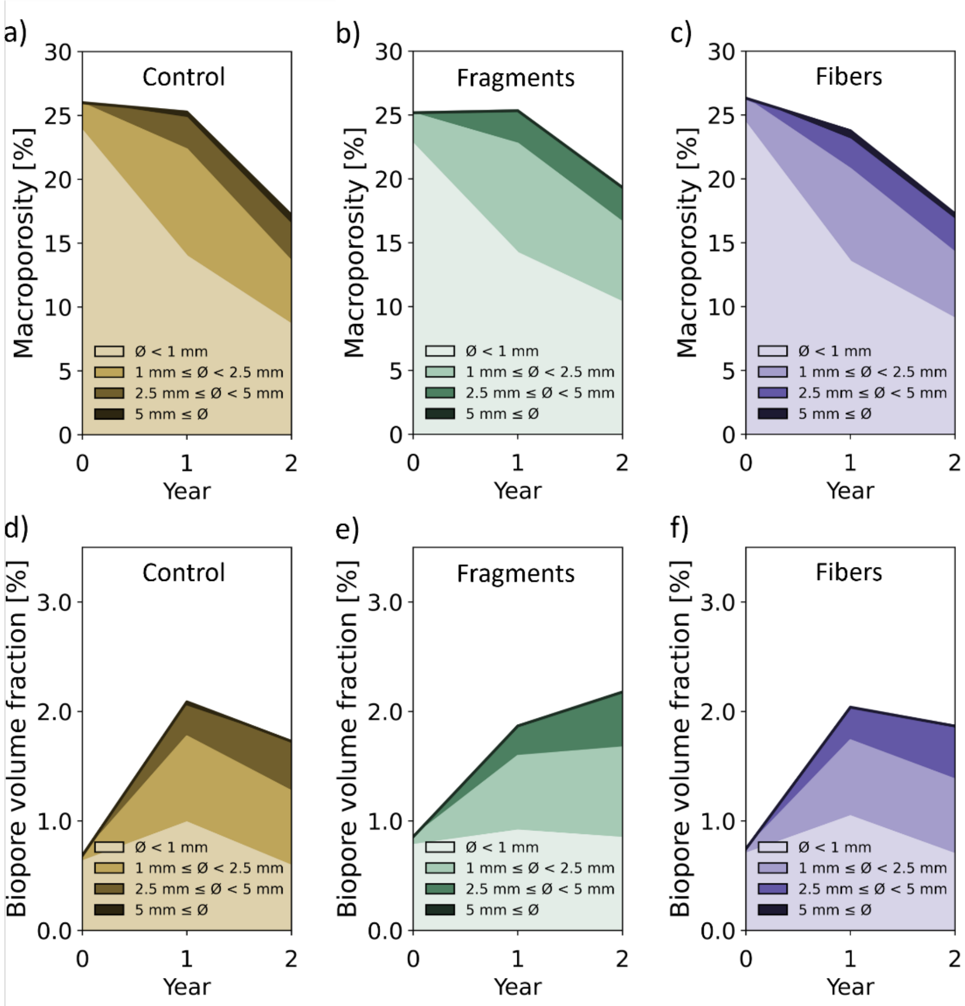

Figure S7: Evolution of size distribution of macropores (panels a-c) and biopores (panel d-f) in ROI 3 over two years.

S5. Temperature and precipitation data

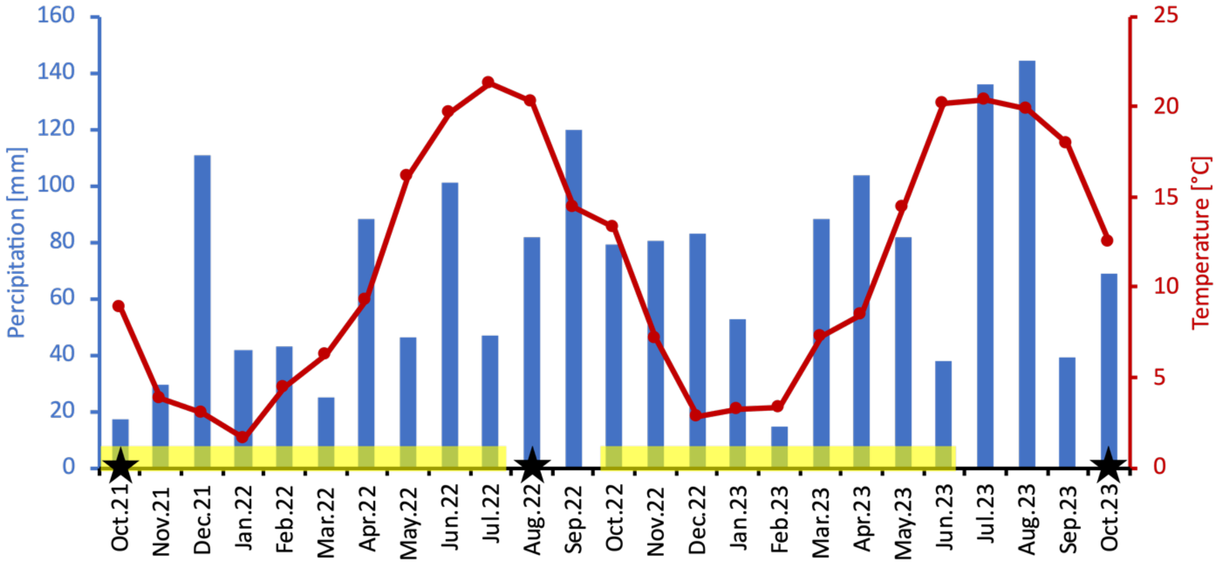

Figure S8: Monthly precipitation (blue) and temperature (red) recorded over two-year incubation. Black stars on the x-axis indicate the sampling time for the x-ray scans and column scarification. Yellow bars on the x-axis show the duration of the winter wheat (Oct. 2021 – Jul. 2022) and barley (Oct. 2022 – Jun. 2023) crops from planting until harvesting.

S6. Column distribution at field site

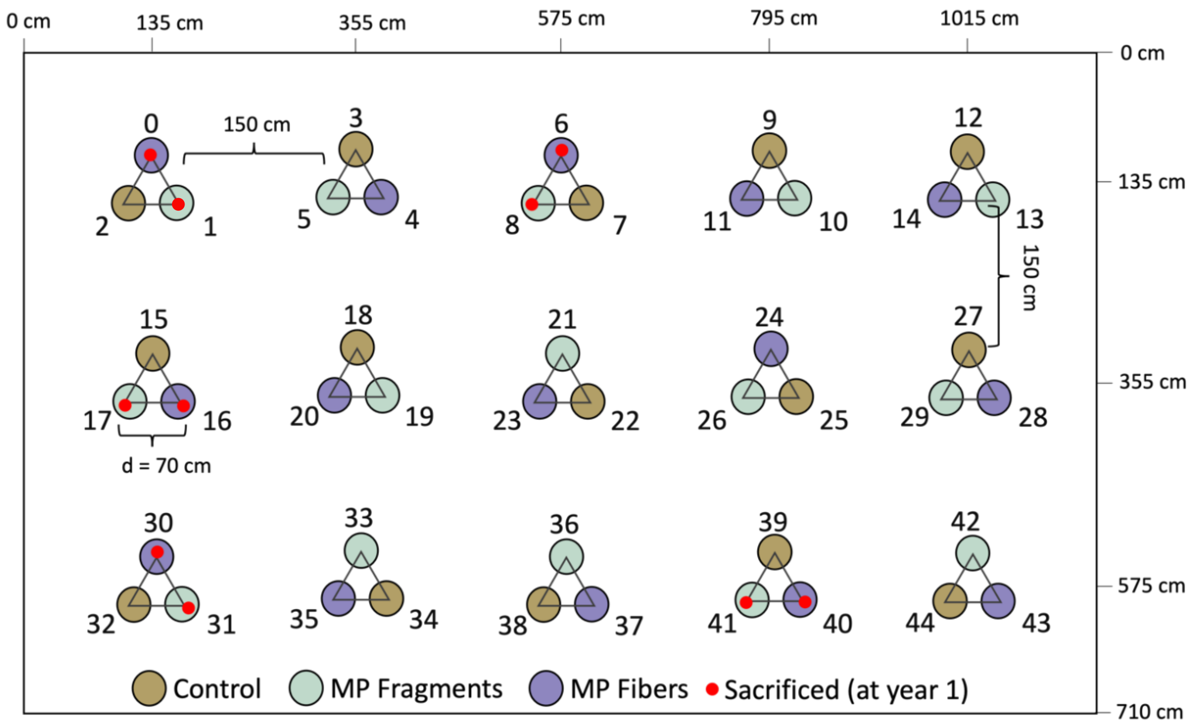

Figure S9: Overview of random column distribution in the field (11 x 7 m) with 15 replicates of control (beige), MPs fragments (cyan) and MPs fibers (violet) treatments. 5 replicates of each MPs treatment containing columns were randomly selected and sacrificed (red dots) for vertical transport analysis after year 1.

S7. Photographs of the field

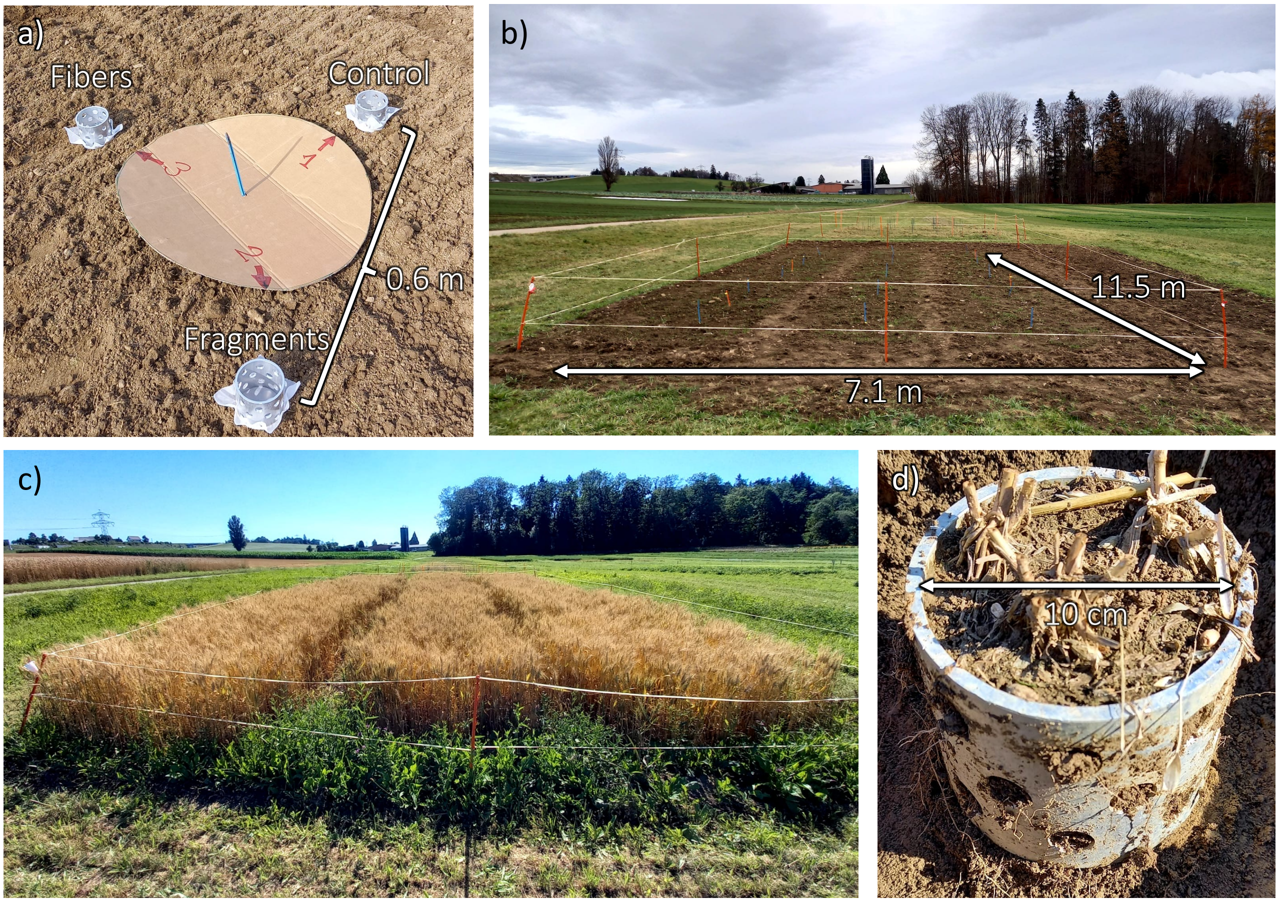

Figure S10: Photographs of the field experiment: (a) arrangement of different treatments in field, (b) field site (December 2021) with developing winter wheat crop, (c) field site (July 2022) with winter wheat shortly before harvesting the crop, (d) excavated column after 1-year incubation (August 2022).

S8. X-ray scanner setup and parameters

Table S1: X-ray scanner setup and reconstruction parameters. Scanner parameters were different at year 0 and year 1 & 2, since there was a different scanner employed at year 0.

**Parameter**	**Used set-up / parameter values**	
Scanning parameter	*Year 0*	*Year 1 & Year 2*
Tube voltage	160 kV	170 kV
Tube current	370 µA	620 µA
Optical filters	-	1 mm Cu
Number of radiographs per 3-D image	1710	2000
Voxel edge length	98.9 µm	114.8 µm
Image processing parameters		
Median filter radius	2 voxels	
Unsharp mask	2 voxels	
Weighing factor of unsharp mask	0.6	
Lower reference value for grey-scale calibration	0.1 percentile of grey values inside column walls	
Upper reference value for grey-scale calibration	Grey values of walls	

S9. Histograms of the grey-values from three-dimensional X-ray images

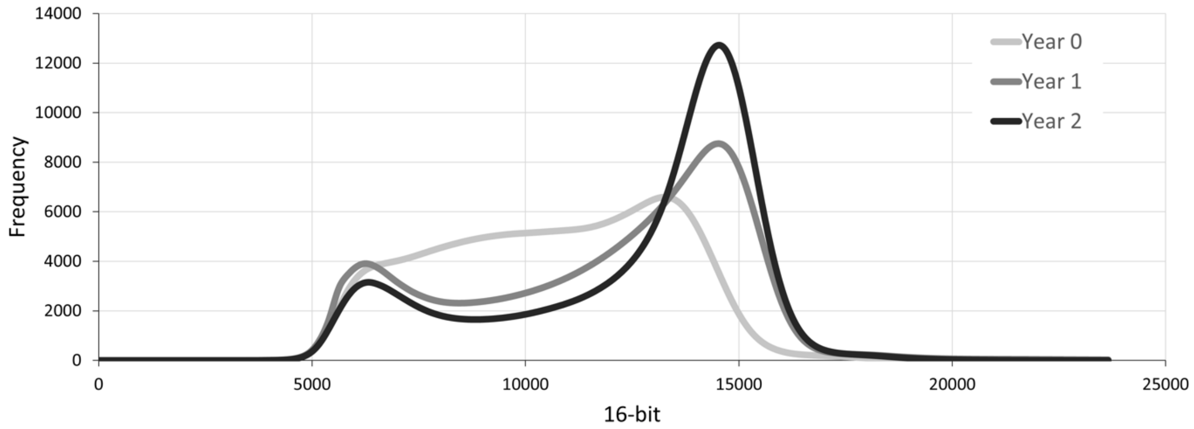

Figure S11: Two-dimensional histograms of the calibrated grey-values extracted from all three-dimensional X-ray images for year 0, year 1 and year 2.

S10 Impact of MPs on crop yield of wheat and barley

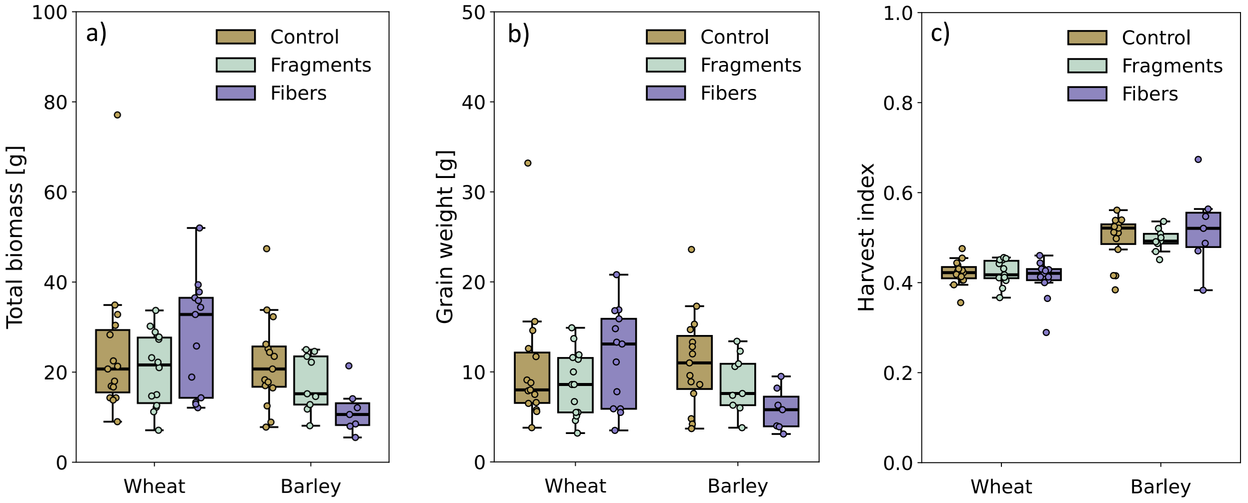

Figure S12: Impact of MPs on crop yield for control (beige), fragment (cyan) and fiber (violet). Panels showing: Total biomass (a), grain weight (b) and harvest index (ratio of grain weight/total biomass) (c). Dots: individual data points, line: median, box: lower and upper quartile, whisker: highest and lowest value, dots outside whisker: outlier. Statistical significance is indicated as follows: p-value <0.001 ***, <0.01 **, and <0.05 *.

S11. Statistical Analysis

Table S2.1: Summary of statistical analyses from a three-way ANOVA assessing the effects of replicate, ROI, year, and their interactions on macroporosity. The table includes information on sums of squares, mean squares, degrees of freedom, F-values, and p-values for each main effect and interaction. Statistical significance is indicated as follows: p-value <0.001 ***, <0.01 **, <0.05 *, and <0.1 •.

**Macroporosity**							
**ANOVA Results**							
	**Sum Sq**	**Mean Sq**	**NumDF**	**DenDF**	**F value**	**Pr(>F)**	**Significance**
Replicate	7.98E+01	3.99E+01	2	2.64E+02	7.92E+00	4.57E-04	***
ROI	2.01E+03	1.00E+03	2	2.87E+01	1.99E+02	1.49E-17	***
Year	8.92E+02	4.46E+02	2	2.47E+01	8.85E+01	5.30E-12	***
Replicate:ROI	5.20E+01	1.30E+01	4	2.59E+02	2.58E+00	3.77E-02	*
Replicate:Year	1.20E+02	3.00E+01	4	2.66E+02	5.96E+00	1.33E-04	***
ROI:Year	7.47E+02	1.87E+02	4	2.60E+02	3.71E+01	1.58E-24	***
Replicate:ROI:Year	4.16E+01	5.20E+00	8	2.53E+02	1.03E+00	4.13E-01	
**Scaled Residuals**							
	**Min.**	**1st Qu.**	**Median**	**Mean**	**3rd Qu.**	**Max.**	
**Freq**	-4.34E+00	-5.79E-01	7.16E-02	2.42E-18	5.93E-01	3.32E+00	
**Random Effects**							
**grp**	**var1**	**vcov**	**sdcor**	**Std.Dev.**			
Block:ROI	(Intercept)	2.21E-01	4.71E-01	4.71E-01			
Block:Year	(Intercept)	4.53E-01	6.73E-01	6.73E-01			
Block	(Intercept)	4.86E-01	6.97E-01	6.97E-01			
Residual		5.04E+00	2.24E+00	2.24E+00			
**Fixed Effects**							
	**Estimate**	**Std. Error**	**df**	**t value**	**Pr(>|t|)**	**Significance**	
(Intercept)	2.42E+01	6.43E-01	245	3.77E+01	6.11E-104	***	
ReplicateFibers	1.10E+00	8.35E-01	250	1.31E+00	1.90E-01		
ReplicateFragments	2.74E-03	8.35E-01	250	3.28E-03	9.97E-01		
ROIROI2	-8.71E+00	8.37E-01	263	-1.04E+01	1.81E-21	***	
ROIROI3	1.76E+00	8.37E-01	263	2.10E+00	3.66E-02	*	
YearYear 1	-6.05E-01	8.56E-01	242	-7.07E-01	4.80E-01		
YearYear 2	-7.02E+00	8.56E-01	242	-8.21E+00	1.32E-14	***	
ReplicateFibers:ROIROI2	3.67E+00	1.19E+00	251	3.08E+00	2.31E-03	**	
ReplicateFragments:ROIROI2	2.82E+00	1.19E+00	251	2.37E+00	1.88E-02	*	
ReplicateFibers:ROIROI3	-7.35E-01	1.21E+00	252	-6.09E-01	5.43E-01		
ReplicateFragments:ROIROI3	-4.16E-01	1.21E+00	252	-3.44E-01	7.31E-01		
ReplicateFibers:YearYear 1	-2.37E+00	1.18E+00	249	-2.01E+00	4.56E-02	*	
ReplicateFragments:YearYear 1	3.75E-01	1.19E+00	251	3.14E-01	7.53E-01		
ReplicateFibers:YearYear 2	-7.37E-01	1.27E+00	261	-5.79E-01	5.63E-01		
ReplicateFragments:YearYear 2	2.17E+00	1.27E+00	261	1.70E+00	8.97E-02	.	
ROIROI2:YearYear 1	-2.27E-01	1.16E+00	248	-1.96E-01	8.45E-01		
ROIROI3:YearYear 1	-1.29E-01	1.16E+00	248	-1.11E-01	9.12E-01		
ROIROI2:YearYear 2	7.16E+00	1.16E+00	248	6.18E+00	2.67E-09	***	
ROIROI3:YearYear 2	-1.78E+00	1.16E+00	248	-1.53E+00	1.27E-01		
ReplicateFibers:ROIROI2:YearYear 1	-1.96E+00	1.68E+00	248	-1.17E+00	2.45E-01		
ReplicateFragments:ROIROI2:YearYear 1	-2.40E+00	1.69E+00	249	-1.42E+00	1.56E-01		
ReplicateFibers:ROIROI3:YearYear 1	1.16E+00	1.70E+00	249	6.84E-01	4.94E-01		
ReplicateFragments:ROIROI3:YearYear 1	8.17E-01	1.71E+00	249	4.77E-01	6.34E-01		
ReplicateFibers:ROIROI2:YearYear 2	-3.28E+00	1.82E+00	256	-1.80E+00	7.27E-02	.	
ReplicateFragments:ROIROI2:YearYear 2	-3.08E+00	1.80E+00	255	-1.72E+00	8.74E-02	.	
ReplicateFibers:ROIROI3:YearYear 2	5.17E-01	1.81E+00	256	2.86E-01	7.75E-01		
ReplicateFragments:ROIROI3:YearYear 2	4.59E-01	1.81E+00	256	2.54E-01	7.99E-01		

Table S2.2: Post-hoc comparisons for macroporosity showing pairwise differences across replicate, ROI, and years. Compairing between replicates within each ROI and year, and temporal differences across years within each replicate and ROI. Results include estimates, standard errors (SE), degrees of freedom (df), t-ratios, p-values, and significance levels, highlighting significant differences over time and between groups. Statistical significance is indicated as follows: p-value <0.001 ***, <0.01 **, <0.05 *, and <0.1 •.

**Macroporosity**								
**Post-hoc Comparisons**								
**Contrast**	**ROI**	**Year**	**Estimate**	**SE**	**df**	**t.ratio**	**p.value**	**Significance**
Control - Fibers	ROI1	Year 0	-1.10E+00	8.36E-01	253	-1.31E+00	3.89E-01	
Control - Fragments	ROI1	Year 0	-2.74E-03	8.36E-01	253	-3.28E-03	1.00E+00	
Fibers - Fragments	ROI1	Year 0	1.10E+00	8.52E-01	256	1.29E+00	4.05E-01	
Control - Fibers	ROI2	Year 0	-4.77E+00	8.54E-01	256	-5.58E+00	1.79E-07	***
Control - Fragments	ROI2	Year 0	-2.82E+00	8.54E-01	256	-3.31E+00	3.08E-03	**
Fibers - Fragments	ROI2	Year 0	1.95E+00	8.89E-01	262	2.19E+00	7.49E-02	.
Control - Fibers	ROI3	Year 0	-3.63E-01	8.75E-01	258	-4.15E-01	9.09E-01	
Control - Fragments	ROI3	Year 0	4.13E-01	8.75E-01	258	4.72E-01	8.85E-01	
Fibers - Fragments	ROI3	Year 0	7.76E-01	9.30E-01	268	8.34E-01	6.82E-01	
Control - Fibers	ROI1	Year 1	1.27E+00	8.36E-01	253	1.52E+00	2.81E-01	
Control - Fragments	ROI1	Year 1	-3.78E-01	8.54E-01	256	-4.43E-01	8.98E-01	
Fibers - Fragments	ROI1	Year 1	-1.65E+00	8.70E-01	259	-1.90E+00	1.41E-01	
Control - Fibers	ROI2	Year 1	-4.42E-01	8.36E-01	253	-5.29E-01	8.57E-01	
Control - Fragments	ROI2	Year 1	-7.99E-01	8.36E-01	253	-9.56E-01	6.05E-01	
Fibers - Fragments	ROI2	Year 1	-3.57E-01	8.52E-01	256	-4.19E-01	9.08E-01	
Control - Fibers	ROI3	Year 1	8.48E-01	8.54E-01	256	9.93E-01	5.82E-01	
Control - Fragments	ROI3	Year 1	-7.79E-01	8.75E-01	258	-8.90E-01	6.47E-01	
Fibers - Fragments	ROI3	Year 1	-1.63E+00	9.09E-01	265	-1.79E+00	1.75E-01	
Control - Fibers	ROI1	Year 2	-3.61E-01	9.63E-01	273	-3.75E-01	9.25E-01	
Control - Fragments	ROI1	Year 2	-2.17E+00	9.63E-01	273	-2.25E+00	6.45E-02	.
Fibers - Fragments	ROI1	Year 2	-1.81E+00	1.07E+00	259	-1.70E+00	2.08E-01	
Control - Fibers	ROI2	Year 2	-7.57E-01	1.00E+00	275	-7.55E-01	7.31E-01	
Control - Fragments	ROI2	Year 2	-1.91E+00	9.63E-01	273	-1.98E+00	1.19E-01	
Fibers - Fragments	ROI2	Year 2	-1.15E+00	1.10E+00	263	-1.04E+00	5.50E-01	
Control - Fibers	ROI3	Year 2	-1.43E-01	9.63E-01	273	-1.49E-01	9.88E-01	
Control - Fragments	ROI3	Year 2	-2.21E+00	9.63E-01	273	-2.30E+00	5.78E-02	.
Fibers - Fragments	ROI3	Year 2	-2.07E+00	1.07E+00	259	-1.94E+00	1.29E-01	
**Contrast**	**Replicate**	**ROI**	**Estimate**	**SE**	**df**	**t.ratio**	**p.value**	**Significance**
Year 0 - Year 1	Control	ROI1	6.05E-01	8.56E-01	246	7.07E-01	7.59E-01	
Year 0 - Year 2	Control	ROI1	7.02E+00	8.56E-01	246	8.21E+00	9.53E-14	***
Year 1 - Year 2	Control	ROI1	6.42E+00	8.56E-01	246	7.50E+00	3.46E-12	***
Year 0 - Year 1	Fibers	ROI1	2.98E+00	8.85E-01	250	3.36E+00	2.55E-03	**
Year 0 - Year 2	Fibers	ROI1	7.76E+00	1.01E+00	268	7.71E+00	9.69E-13	***
Year 1 - Year 2	Fibers	ROI1	4.78E+00	1.01E+00	268	4.75E+00	9.73E-06	***
Year 0 - Year 1	Fragments	ROI1	2.30E-01	9.02E-01	254	2.55E-01	9.65E-01	
Year 0 - Year 2	Fragments	ROI1	4.86E+00	1.01E+00	268	4.83E+00	6.96E-06	***
Year 1 - Year 2	Fragments	ROI1	4.63E+00	1.02E+00	271	4.53E+00	2.68E-05	***
Year 0 - Year 1	Control	ROI2	8.32E-01	8.56E-01	246	9.73E-01	5.95E-01	
Year 0 - Year 2	Control	ROI2	-1.34E-01	8.56E-01	246	-1.57E-01	9.87E-01	
Year 1 - Year 2	Control	ROI2	-9.67E-01	8.56E-01	246	-1.13E+00	4.97E-01	
Year 0 - Year 1	Fibers	ROI2	5.16E+00	9.02E-01	254	5.72E+00	8.99E-08	***
Year 0 - Year 2	Fibers	ROI2	3.88E+00	1.06E+00	277	3.66E+00	8.85E-04	***
Year 1 - Year 2	Fibers	ROI2	-1.28E+00	1.04E+00	273	-1.23E+00	4.38E-01	
Year 0 - Year 1	Fragments	ROI2	2.86E+00	9.02E-01	254	3.17E+00	4.89E-03	**
Year 0 - Year 2	Fragments	ROI2	7.82E-01	1.02E+00	272	7.65E-01	7.25E-01	
Year 1 - Year 2	Fragments	ROI2	-2.08E+00	1.01E+00	268	-2.06E+00	9.95E-02	.
Year 0 - Year 1	Control	ROI3	7.34E-01	8.56E-01	246	8.57E-01	6.68E-01	
Year 0 - Year 2	Control	ROI3	8.80E+00	8.56E-01	246	1.03E+01	5.51E-14	***
Year 1 - Year 2	Control	ROI3	8.07E+00	8.56E-01	246	9.43E+00	5.65E-14	***
Year 0 - Year 1	Fibers	ROI3	1.94E+00	9.37E-01	258	2.08E+00	9.70E-02	.
Year 0 - Year 2	Fibers	ROI3	9.02E+00	1.04E+00	276	8.66E+00	3.22E-13	***
Year 1 - Year 2	Fibers	ROI3	7.07E+00	1.02E+00	272	6.92E+00	9.82E-11	***
Year 0 - Year 1	Fragments	ROI3	-4.58E-01	9.55E-01	258	-4.80E-01	8.81E-01	
Year 0 - Year 2	Fragments	ROI3	6.17E+00	1.04E+00	276	5.93E+00	2.71E-08	***
Year 1 - Year 2	Fragments	ROI3	6.63E+00	1.04E+00	276	6.37E+00	2.37E-09	***

Table S3.1: Summary of statistical analyses from a three-way ANOVA assessing the effects of replicate, ROI, year, and their interactions on macropore surface area. The table includes information on sums of squares, mean squares, degrees of freedom, F-values, and p-values for each main effect and interaction. Statistical significance is indicated as follows: p-value <0.001 ***, <0.01 **, <0.05 *, and <0.1 •.

Macropore Surface Area							
**ANOVA Results**							
	**Sum Sq**	**Mean Sq**	**NumDF**	**DenDF**	**F value**	**Pr(>F)**	**Significance**
Replicate	2.43E-03	1.22E-03	2	2.61E+02	3.77E+00	2.43E-02	*
ROI	2.02E+00	1.01E+00	2	3.02E+01	3.14E+03	1.03E-35	***
Year	3.14E-01	1.57E-01	2	2.57E+01	4.87E+02	3.72E-21	***
Replicate:ROI	5.00E-04	1.25E-04	4	2.57E+02	3.87E-01	8.18E-01	
Replicate:Year	1.09E-02	2.72E-03	4	2.63E+02	8.42E+00	2.10E-06	***
ROI:Year	2.81E-01	7.02E-02	4	2.56E+02	2.18E+02	4.18E-81	***
Replicate:ROI:Year	7.88E-03	9.86E-04	8	2.51E+02	3.06E+00	2.67E-03	**
**Scaled Residuals**							
	**Min.**	**1st Qu.**	**Median**	**Mean**	**3rd Qu.**	**Max.**	
**Freq**	-3.26E+00	-5.58E-01	-2.52E-02	-1.98E-17	5.52E-01	3.72E+00	
**Random Effects**							
**grp**	**var1**	**vcov**	**sdcor**	**Std.Dev.**			
Block:ROI	(Intercept)	3.42E-05	5.85E-03	5.85E-03			
Block:Year	(Intercept)	5.49E-05	7.41E-03	7.41E-03			
Block	(Intercept)	7.07E-05	8.41E-03	8.41E-03			
Residual		3.23E-04	1.80E-02	1.80E-02			
**Fixed Effects**							
	**Estimate**	**Std. Error**	**df**	**t value**	**Pr(>|t|)**	**Significance**	
(Intercept)	4.05E-01	5.67E-03	159	7.14E+01	1.12E-122	***	
ReplicateFibers	1.97E-02	6.69E-03	249	2.94E+00	3.57E-03	**	
ReplicateFragments	-3.59E-03	6.69E-03	249	-5.36E-01	5.92E-01		
ROIROI2	-3.55E-01	6.90E-03	239	-5.15E+01	2.00E-131	***	
ROIROI3	-1.34E-01	6.90E-03	239	-1.94E+01	7.08E-51	***	
YearYear 1	-1.25E-01	7.09E-03	207	-1.76E+01	6.04E-43	***	
YearYear 2	-1.99E-01	7.09E-03	207	-2.81E+01	1.38E-72	***	
ReplicateFibers:ROIROI2	-7.42E-03	9.45E-03	248	-7.85E-01	4.33E-01		
ReplicateFragments:ROIROI2	1.45E-02	9.68E-03	250	1.50E+00	1.35E-01		
ReplicateFibers:ROIROI3	-1.98E-02	9.56E-03	249	-2.08E+00	3.89E-02	*	
ReplicateFragments:ROIROI3	-1.36E-02	9.45E-03	248	-1.44E+00	1.52E-01		
ReplicateFibers:YearYear 1	-3.92E-02	9.68E-03	250	-4.05E+00	6.85E-05	***	
ReplicateFragments:YearYear 1	2.72E-03	9.45E-03	248	2.88E-01	7.73E-01		
ReplicateFibers:YearYear 2	6.23E-03	1.05E-02	260	5.95E-01	5.52E-01		
ReplicateFragments:YearYear 2	3.35E-02	1.02E-02	259	3.28E+00	1.18E-03	**	
ROIROI2:YearYear 1	1.14E-01	9.28E-03	247	1.23E+01	2.08E-27	***	
ROIROI3:YearYear 1	3.50E-02	9.28E-03	247	3.78E+00	1.98E-04	***	
ROIROI2:YearYear 2	1.86E-01	9.38E-03	249	1.98E+01	3.61E-53	***	
ROIROI3:YearYear 2	4.74E-02	9.37E-03	248	5.06E+00	8.23E-07	***	
ReplicateFibers:ROIROI2:YearYear 1	2.88E-02	1.37E-02	249	2.11E+00	3.60E-02	*	
ReplicateFragments:ROIROI2:YearYear 1	-1.28E-02	1.35E-02	248	-9.50E-01	3.43E-01		
ReplicateFibers:ROIROI3:YearYear 1	3.24E-02	1.36E-02	249	2.38E+00	1.80E-02	*	
ReplicateFragments:ROIROI3:YearYear 1	1.59E-02	1.34E-02	247	1.18E+00	2.39E-01		
ReplicateFibers:ROIROI2:YearYear 2	-1.37E-02	1.47E-02	253	-9.32E-01	3.52E-01		
ReplicateFragments:ROIROI2:YearYear 2	-4.11E-02	1.45E-02	252	-2.83E+00	5.10E-03	**	
ReplicateFibers:ROIROI3:YearYear 2	7.26E-03	1.50E-02	254	4.84E-01	6.29E-01		
ReplicateFragments:ROIROI3:YearYear 2	9.69E-03	1.44E-02	252	6.73E-01	5.02E-01		

Table S3.2: Post-hoc comparisons for macropore surface area showing pairwise differences across replicate, ROI, and years. Compairing between replicates within each ROI and year, and temporal differences across years within each replicate and ROI. Results include estimates, standard errors (SE), degrees of freedom (df), t-ratios, p-values, and significance levels, highlighting significant differences over time and between groups. Statistical significance is indicated as follows: p-value <0.001 ***, <0.01 **, <0.05 *, and <0.1 •.

Macropore Surface Area								
Post-hoc Comparisons								
Contrast	ROI	Year	Estimate	SE	df	t.ratio	p.value	Significance
Control - Fibers	ROI1	Year 0	-1.97E-02	6.69E-03	249	-2.94E+00	9.97E-03	**
Control - Fragments	ROI1	Year 0	3.59E-03	6.69E-03	249	5.36E-01	8.54E-01	
Fibers - Fragments	ROI1	Year 0	2.33E-02	6.83E-03	251	3.41E+00	2.19E-03	**
Control - Fibers	ROI2	Year 0	-1.23E-02	6.69E-03	249	-1.83E+00	1.61E-01	
Control - Fragments	ROI2	Year 0	-1.09E-02	7.02E-03	253	-1.56E+00	2.65E-01	
Fibers - Fragments	ROI2	Year 0	1.32E-03	7.15E-03	256	1.85E-01	9.81E-01	
Control - Fibers	ROI3	Year 0	1.47E-04	6.84E-03	251	2.15E-02	1.00E+00	
Control - Fragments	ROI3	Year 0	1.72E-02	6.69E-03	249	2.57E+00	2.92E-02	*
Fibers - Fragments	ROI3	Year 0	1.70E-02	6.98E-03	253	2.44E+00	4.06E-02	*
Control - Fibers	ROI1	Year 1	1.95E-02	7.02E-03	253	2.78E+00	1.60E-02	*
Control - Fragments	ROI1	Year 1	8.63E-04	6.69E-03	249	1.29E-01	9.91E-01	
Fibers - Fragments	ROI1	Year 1	-1.87E-02	7.15E-03	256	-2.61E+00	2.61E-02	*
Control - Fibers	ROI2	Year 1	-1.87E-03	7.02E-03	253	-2.67E-01	9.61E-01	
Control - Fragments	ROI2	Year 1	-8.26E-04	6.69E-03	249	-1.23E-01	9.92E-01	
Fibers - Fragments	ROI2	Year 1	1.05E-03	7.15E-03	256	1.46E-01	9.88E-01	
Control - Fibers	ROI3	Year 1	6.98E-03	6.69E-03	249	1.04E+00	5.50E-01	
Control - Fragments	ROI3	Year 1	-1.40E-03	6.84E-03	251	-2.04E-01	9.77E-01	
Fibers - Fragments	ROI3	Year 1	-8.38E-03	6.98E-03	253	-1.20E+00	4.54E-01	
Control - Fibers	ROI1	Year 2	-2.59E-02	8.09E-03	268	-3.20E+00	4.33E-03	**
Control - Fragments	ROI1	Year 2	-2.99E-02	7.76E-03	266	-3.86E+00	4.23E-04	***
Fibers - Fragments	ROI1	Year 2	-4.02E-03	8.86E-03	257	-4.54E-01	8.93E-01	
Control - Fibers	ROI2	Year 2	-4.79E-03	8.17E-03	266	-5.86E-01	8.28E-01	
Control - Fragments	ROI2	Year 2	-3.41E-03	7.84E-03	264	-4.35E-01	9.01E-01	
Fibers - Fragments	ROI2	Year 2	1.38E-03	8.86E-03	258	1.56E-01	9.87E-01	
Control - Fibers	ROI3	Year 2	-1.33E-02	8.57E-03	270	-1.56E+00	2.66E-01	
Control - Fragments	ROI3	Year 2	-2.60E-02	7.91E-03	271	-3.29E+00	3.23E-03	**
Fibers - Fragments	ROI3	Year 2	-1.27E-02	9.24E-03	262	-1.37E+00	3.56E-01	
Contrast	Replicate	ROI	Estimate	SE	df	t.ratio	p.value	Significance
Year 0 - Year 1	Control	ROI1	1.25E-01	7.09E-03	212	1.76E+01	6.78E-14	***
Year 0 - Year 2	Control	ROI1	1.99E-01	7.09E-03	212	2.81E+01	6.78E-14	***
Year 1 - Year 2	Control	ROI1	7.47E-02	7.09E-03	212	1.05E+01	9.18E-14	***
Year 0 - Year 1	Fibers	ROI1	1.64E-01	7.62E-03	230	2.15E+01	0.00E+00	***
Year 0 - Year 2	Fibers	ROI1	1.93E-01	8.63E-03	250	2.24E+01	1.26E-13	***
Year 1 - Year 2	Fibers	ROI1	2.92E-02	8.87E-03	255	3.29E+00	3.22E-03	**
Year 0 - Year 1	Fragments	ROI1	1.22E-01	7.33E-03	219	1.67E+01	4.51E-14	***
Year 0 - Year 2	Fragments	ROI1	1.66E-01	8.32E-03	243	1.99E+01	3.89E-14	***
Year 1 - Year 2	Fragments	ROI1	4.39E-02	8.32E-03	243	5.27E+00	8.83E-07	***
Year 0 - Year 1	Control	ROI2	1.08E-02	7.09E-03	212	1.52E+00	2.85E-01	
Year 0 - Year 2	Control	ROI2	1.35E-02	7.23E-03	215	1.87E+00	1.51E-01	
Year 1 - Year 2	Control	ROI2	2.74E-03	7.23E-03	215	3.78E-01	9.24E-01	
Year 0 - Year 1	Fibers	ROI2	2.12E-02	7.63E-03	230	2.77E+00	1.64E-02	*
Year 0 - Year 2	Fibers	ROI2	2.10E-02	8.63E-03	251	2.43E+00	4.15E-02	*
Year 1 - Year 2	Fibers	ROI2	-1.79E-04	8.85E-03	252	-2.02E-02	1.00E+00	
Year 0 - Year 1	Fragments	ROI2	2.09E-02	7.62E-03	230	2.74E+00	1.82E-02	*
Year 0 - Year 2	Fragments	ROI2	2.10E-02	8.58E-03	249	2.45E+00	3.93E-02	*
Year 1 - Year 2	Fragments	ROI2	1.55E-04	8.32E-03	243	1.86E-02	1.00E+00	
Year 0 - Year 1	Control	ROI3	8.97E-02	7.09E-03	212	1.26E+01	6.79E-14	***
Year 0 - Year 2	Control	ROI3	1.52E-01	7.22E-03	217	2.11E+01	0.00E+00	***
Year 1 - Year 2	Control	ROI3	6.23E-02	7.22E-03	217	8.63E+00	1.19E-14	***
Year 0 - Year 1	Fibers	ROI3	9.66E-02	7.47E-03	224	1.29E+01	0.00E+00	***
Year 0 - Year 2	Fibers	ROI3	1.39E-01	9.13E-03	262	1.52E+01	6.78E-14	***
Year 1 - Year 2	Fibers	ROI3	4.20E-02	9.00E-03	259	4.66E+00	1.48E-05	***
Year 0 - Year 1	Fragments	ROI3	7.11E-02	7.47E-03	224	9.53E+00	9.60E-14	***
Year 0 - Year 2	Fragments	ROI3	1.09E-01	8.32E-03	243	1.31E+01	8.24E-14	***
Year 1 - Year 2	Fragments	ROI3	3.77E-02	8.45E-03	247	4.46E+00	3.70E-05	***

Table S4.1: Summary of statistical analyses from a three-way ANOVA assessing the effects of replicate, ROI, year, and their interactions on critical macropore diameter. The table includes information on sums of squares, mean squares, degrees of freedom, F-values, and p-values for each main effect and interaction. Statistical significance is indicated as follows: p-value <0.001 ***, <0.01 **, <0.05 *, and <0.1 •.

Critical Pore Diameter							
**ANOVA Results**							
	**Sum Sq**	**Mean Sq**	**NumDF**	**DenDF**	**F value**	**Pr(>F)**	**Significance**
Replicate	4.34E-01	2.17E-01	2	3.17E+02	8.37E-01	4.34E-01	
ROI	4.63E+01	2.32E+01	2	3.17E+02	8.93E+01	1.72E-31	***
Year	7.22E+01	3.61E+01	2	3.17E+02	1.39E+02	4.20E-44	***
Replicate:ROI	1.46E+00	3.66E-01	4	3.17E+02	1.41E+00	2.31E-01	
Replicate:Year	9.66E-01	2.42E-01	4	3.17E+02	9.31E-01	4.46E-01	
ROI:Year	2.38E+01	5.95E+00	4	3.17E+02	2.29E+01	1.17E-16	***
Replicate:ROI:Year	2.19E+00	2.74E-01	8	3.17E+02	1.06E+00	3.95E-01	
**Scaled Residuals**							
	**Min.**	**1st Qu.**	**Median**	**Mean**	**3rd Qu.**	**Max.**	
**Freq**	-2.92E+00	-3.82E-01	-4.39E-02	-2.00E-18	1.97E-01	3.91E+00	
**Random Effects**							
**grp**	**var1**	**vcov**	**sdcor**	**Std.Dev.**			
Block:ROI	(Intercept)	0	0	0			
Block:Year	(Intercept)	0	0	0			
Block	(Intercept)	0	0	0			
Residual		2.59E-01	5.09E-01	5.09E-01			
**Fixed Effects**							
	**Estimate**	**Std. Error**	**df**	**t value**	**Pr(>|t|)**	**Significance**	
(Intercept)	4.12E-01	1.31E-01	317	3.14E+00	1.87E-03	**	
ReplicateFibers	2.70E-02	1.93E-01	317	1.40E-01	8.89E-01		
ReplicateFragments	-1.07E-02	1.89E-01	317	-5.68E-02	9.55E-01		
ROIROI2	8.24E-02	1.86E-01	317	4.43E-01	6.58E-01		
ROIROI3	1.12E-01	1.86E-01	317	6.05E-01	5.46E-01		
YearYear 1	3.96E-01	1.86E-01	317	2.13E+00	3.39E-02	*	
YearYear 2	3.64E-01	1.86E-01	317	1.96E+00	5.13E-02	.	
ReplicateFibers:ROIROI2	-1.94E-03	2.76E-01	317	-7.03E-03	9.94E-01		
ReplicateFragments:ROIROI2	-2.19E-03	2.70E-01	317	-8.11E-03	9.94E-01		
ReplicateFibers:ROIROI3	-4.50E-02	2.70E-01	317	-1.67E-01	8.68E-01		
ReplicateFragments:ROIROI3	3.84E-02	2.68E-01	317	1.43E-01	8.86E-01		
ReplicateFibers:YearYear 1	-3.47E-02	2.73E-01	317	-1.27E-01	8.99E-01		
ReplicateFragments:YearYear 1	8.32E-02	2.68E-01	317	3.11E-01	7.56E-01		
ReplicateFibers:YearYear 2	1.63E-01	2.95E-01	317	5.53E-01	5.81E-01		
ReplicateFragments:YearYear 2	-4.42E-02	2.92E-01	317	-1.51E-01	8.80E-01		
ROIROI2:YearYear 1	1.09E+00	2.63E-01	317	4.13E+00	4.64E-05	***	
ROIROI3:YearYear 1	3.74E-01	2.63E-01	317	1.42E+00	1.56E-01		
ROIROI2:YearYear 2	1.33E+00	2.63E-01	317	5.05E+00	7.62E-07	***	
ROIROI3:YearYear 2	4.05E-01	2.63E-01	317	1.54E+00	1.25E-01		
ReplicateFibers:ROIROI2:YearYear 1	-7.95E-02	3.86E-01	317	-2.06E-01	8.37E-01		
ReplicateFragments:ROIROI2:YearYear 1	1.75E-01	3.80E-01	317	4.61E-01	6.45E-01		
ReplicateFibers:ROIROI3:YearYear 1	4.30E-01	3.82E-01	317	1.12E+00	2.62E-01		
ReplicateFragments:ROIROI3:YearYear 1	5.45E-02	3.78E-01	317	1.44E-01	8.86E-01		
ReplicateFibers:ROIROI2:YearYear 2	1.58E-03	4.32E-01	317	3.65E-03	9.97E-01		
ReplicateFragments:ROIROI2:YearYear 2	3.80E-01	4.21E-01	317	9.03E-01	3.67E-01		
ReplicateFibers:ROIROI3:YearYear 2	-5.23E-02	4.11E-01	317	-1.27E-01	8.99E-01		
ReplicateFragments:ROIROI3:YearYear 2	-5.17E-01	4.14E-01	317	-1.25E+00	2.12E-01		

Table S4.2: Post-hoc comparisons for critical macropore diameter showing pairwise differences across replicate, ROI, and years. Compairing between replicates within each ROI and year, and temporal differences across years within each replicate and ROI. Results include estimates, standard errors (SE), degrees of freedom (df), t-ratios, p-values, and significance levels, highlighting significant differences over time and between groups. Statistical significance is indicated as follows: p-value <0.001 ***, <0.01 **, <0.05 *, and <0.1 •.

**Critical Pore Diameter**								
**Post-hoc Comparisons**								
**Contrast**	**ROI**	**Year**	**Estimate**	**SE**	**df**	**t.ratio**	**p.value**	**Significance**
Control - Fibers	ROI1	Year 0	-2.70E-02	1.93E-01	258	-1.40E-01	9.89E-01	
Control - Fragments	ROI1	Year 0	1.07E-02	1.89E-01	254	5.67E-02	9.98E-01	
Fibers - Fragments	ROI1	Year 0	3.77E-02	1.97E-01	262	1.92E-01	9.80E-01	
Control - Fibers	ROI2	Year 0	-2.50E-02	1.98E-01	262	-1.27E-01	9.91E-01	
Control - Fragments	ROI2	Year 0	1.29E-02	1.93E-01	258	6.69E-02	9.98E-01	
Fibers - Fragments	ROI2	Year 0	3.80E-02	2.05E-01	271	1.86E-01	9.81E-01	
Control - Fibers	ROI3	Year 0	1.80E-02	1.89E-01	254	9.53E-02	9.95E-01	
Control - Fragments	ROI3	Year 0	-2.76E-02	1.89E-01	254	-1.46E-01	9.88E-01	
Fibers - Fragments	ROI3	Year 0	-4.57E-02	1.93E-01	258	-2.37E-01	9.69E-01	
Control - Fibers	ROI1	Year 1	7.77E-03	1.93E-01	258	4.02E-02	9.99E-01	
Control - Fragments	ROI1	Year 1	-7.24E-02	1.89E-01	254	-3.83E-01	9.23E-01	
Fibers - Fragments	ROI1	Year 1	-8.02E-02	1.96E-01	262	-4.08E-01	9.12E-01	
Control - Fibers	ROI2	Year 1	8.92E-02	1.89E-01	254	4.71E-01	8.85E-01	
Control - Fragments	ROI2	Year 1	-2.46E-01	1.89E-01	254	-1.30E+00	3.98E-01	
Fibers - Fragments	ROI2	Year 1	-3.35E-01	1.93E-01	258	-1.74E+00	1.93E-01	
Control - Fibers	ROI3	Year 1	-3.77E-01	1.89E-01	254	-1.99E+00	1.16E-01	
Control - Fragments	ROI3	Year 1	-1.65E-01	1.89E-01	254	-8.73E-01	6.58E-01	
Fibers - Fragments	ROI3	Year 1	2.12E-01	1.93E-01	258	1.10E+00	5.16E-01	
Control - Fibers	ROI1	Year 2	-1.90E-01	2.24E-01	281	-8.48E-01	6.74E-01	
Control - Fragments	ROI1	Year 2	5.49E-02	2.24E-01	281	2.45E-01	9.67E-01	
Fibers - Fragments	ROI1	Year 2	2.45E-01	2.56E-01	276	9.58E-01	6.04E-01	
Control - Fibers	ROI2	Year 2	-1.90E-01	2.48E-01	292	-7.65E-01	7.25E-01	
Control - Fragments	ROI2	Year 2	-3.23E-01	2.35E-01	286	-1.38E+00	3.55E-01	
Fibers - Fragments	ROI2	Year 2	-1.33E-01	2.86E-01	288	-4.67E-01	8.87E-01	
Control - Fibers	ROI3	Year 2	-9.28E-02	2.16E-01	276	-4.30E-01	9.03E-01	
Control - Fragments	ROI3	Year 2	5.33E-01	2.24E-01	281	2.38E+00	4.72E-02	*
Fibers - Fragments	ROI3	Year 2	6.26E-01	2.48E-01	269	2.52E+00	3.27E-02	*
**Contrast**	**Replicate**	**ROI**	**Estimate**	**SE**	**df**	**t.ratio**	**p.value**	**Significance**
Year 0 - Year 1	Control	ROI1	-3.96E-01	1.86E-01	273	-2.13E+00	8.56E-02	.
Year 0 - Year 2	Control	ROI1	-3.64E-01	1.86E-01	273	-1.96E+00	1.25E-01	
Year 1 - Year 2	Control	ROI1	3.24E-02	1.86E-01	273	1.74E-01	9.83E-01	
Year 0 - Year 1	Fibers	ROI1	-3.61E-01	2.00E-01	278	-1.81E+00	1.69E-01	
Year 0 - Year 2	Fibers	ROI1	-5.27E-01	2.30E-01	296	-2.29E+00	5.89E-02	.
Year 1 - Year 2	Fibers	ROI1	-1.65E-01	2.30E-01	290	-7.19E-01	7.52E-01	
Year 0 - Year 1	Fragments	ROI1	-4.79E-01	1.93E-01	273	-2.49E+00	3.56E-02	*
Year 0 - Year 2	Fragments	ROI1	-3.20E-01	2.27E-01	292	-1.41E+00	3.38E-01	
Year 1 - Year 2	Fragments	ROI1	1.60E-01	2.27E-01	292	7.04E-01	7.61E-01	
Year 0 - Year 1	Control	ROI2	-1.48E+00	1.86E-01	273	-7.97E+00	5.55E-13	***
Year 0 - Year 2	Control	ROI2	-1.69E+00	1.86E-01	273	-9.09E+00	4.22E-13	***
Year 1 - Year 2	Control	ROI2	-2.08E-01	1.86E-01	273	-1.12E+00	5.02E-01	
Year 0 - Year 1	Fibers	ROI2	-1.37E+00	2.01E-01	278	-6.81E+00	1.76E-10	***
Year 0 - Year 2	Fibers	ROI2	-1.86E+00	2.57E-01	307	-7.22E+00	1.37E-11	***
Year 1 - Year 2	Fibers	ROI2	-4.87E-01	2.50E-01	299	-1.95E+00	1.28E-01	
Year 0 - Year 1	Fragments	ROI2	-1.74E+00	1.96E-01	276	-8.87E+00	3.43E-13	***
Year 0 - Year 2	Fragments	ROI2	-2.03E+00	2.40E-01	294	-8.44E+00	8.55E-13	***
Year 1 - Year 2	Fragments	ROI2	-2.86E-01	2.37E-01	296	-1.20E+00	4.52E-01	
Year 0 - Year 1	Control	ROI3	-7.70E-01	1.86E-01	273	-4.14E+00	1.36E-04	***
Year 0 - Year 2	Control	ROI3	-7.69E-01	1.86E-01	273	-4.13E+00	1.41E-04	***
Year 1 - Year 2	Control	ROI3	1.61E-03	1.86E-01	273	8.65E-03	1.00E+00	
Year 0 - Year 1	Fibers	ROI3	-1.17E+00	1.93E-01	273	-6.05E+00	1.42E-08	***
Year 0 - Year 2	Fibers	ROI3	-8.79E-01	2.18E-01	288	-4.03E+00	2.14E-04	***
Year 1 - Year 2	Fibers	ROI3	2.86E-01	2.18E-01	288	1.31E+00	3.92E-01	
Year 0 - Year 1	Fragments	ROI3	-9.08E-01	1.93E-01	273	-4.71E+00	1.15E-05	***
Year 0 - Year 2	Fragments	ROI3	-2.08E-01	2.27E-01	292	-9.15E-01	6.31E-01	
Year 1 - Year 2	Fragments	ROI3	7.00E-01	2.27E-01	292	3.09E+00	6.27E-03	**

Table S5.1: Summary of statistical analyses from a three-way ANOVA assessing the effects of replicate, ROI, year, and their interactions on connection probability. The table includes information on sums of squares, mean squares, degrees of freedom, F-values, and p-values for each main effect and interaction. Statistical significance is indicated as follows: p-value <0.001 ***, <0.01 **, <0.05 *, and <0.1 •.

**Connection Probability**							
**ANOVA Results**							
	**Sum Sq**	**Mean Sq**	**NumDF**	**DenDF**	**F value**	**Pr(>F)**	**Significance**
Replicate	1.02E-02	5.10E-03	2	2.45E+02	8.50E+00	2.69E-04	***
ROI	1.63E-01	8.15E-02	2	2.77E+01	1.36E+02	4.87E-15	***
Year	4.43E-02	2.22E-02	2	2.51E+01	3.70E+01	3.35E-08	***
Replicate:ROI	5.02E-03	1.25E-03	4	2.37E+02	2.09E+00	8.23E-02	.
Replicate:Year	7.16E-03	1.79E-03	4	2.45E+02	2.99E+00	1.96E-02	*
ROI:Year	8.45E-02	2.11E-02	4	2.36E+02	3.52E+01	4.44E-23	***
Replicate:ROI:Year	2.21E-02	2.76E-03	8	2.32E+02	4.61E+00	3.06E-05	***
**Scaled Residuals**							
	**Min.**	**1st Qu.**	**Median**	**Mean**	**3rd Qu.**	**Max.**	
**Freq**	-4.71E+00	-2.58E-01	1.39E-02	-9.67E-18	3.43E-01	3.25E+00	
**Random Effects**							
**grp**	**var1**	**vcov**	**sdcor**	**Std.Dev.**			
Block:ROI	(Intercept)	1.24E-04	1.12E-02	1.12E-02			
Block:Year	(Intercept)	1.36E-04	1.17E-02	1.17E-02			
Block	(Intercept)	9.59E-07	9.79E-04	9.79E-04			
Residual		5.99E-04	2.45E-02	2.45E-02			
**Fixed Effects**							
	**Estimate**	**Std. Error**	**df**	**t value**	**Pr(>|t|)**	**Significance**	
(Intercept)	9.84E-01	7.80E-03	225	1.26E+02	7.55E-211	***	
ReplicateFibers	6.80E-03	9.69E-03	230	7.02E-01	4.83E-01		
ReplicateFragments	2.39E-03	9.32E-03	232	2.57E-01	7.97E-01		
ROIROI2	-8.93E-02	1.01E-02	199	-8.81E+00	6.08E-16	***	
ROIROI3	1.04E-02	1.02E-02	201	1.02E+00	3.08E-01		
YearYear 1	-1.64E-02	1.02E-02	188	-1.60E+00	1.12E-01		
YearYear 2	-4.41E-02	1.01E-02	183	-4.38E+00	1.98E-05	***	
ReplicateFibers:ROIROI2	6.76E-02	1.37E-02	229	4.95E+00	1.47E-06	***	
ReplicateFragments:ROIROI2	5.42E-02	1.34E-02	231	4.03E+00	7.50E-05	***	
ReplicateFibers:ROIROI3	-7.31E-03	1.36E-02	231	-5.39E-01	5.90E-01		
ReplicateFragments:ROIROI3	-2.48E-03	1.33E-02	231	-1.87E-01	8.52E-01		
ReplicateFibers:YearYear 1	-1.24E-02	1.34E-02	230	-9.20E-01	3.58E-01		
ReplicateFragments:YearYear 1	-3.32E-03	1.33E-02	229	-2.51E-01	8.02E-01		
ReplicateFibers:YearYear 2	1.00E-02	1.51E-02	241	6.63E-01	5.08E-01		
ReplicateFragments:YearYear 2	9.26E-03	1.41E-02	239	6.57E-01	5.12E-01		
ROIROI2:YearYear 1	-3.22E-02	1.33E-02	231	-2.42E+00	1.62E-02	*	
ROIROI3:YearYear 1	2.46E-03	1.31E-02	230	1.87E-01	8.51E-01		
ROIROI2:YearYear 2	4.95E-02	1.32E-02	230	3.76E+00	2.19E-04	***	
ROIROI3:YearYear 2	-1.12E-02	1.29E-02	229	-8.71E-01	3.85E-01		
ReplicateFibers:ROIROI2:YearYear 1	-4.96E-02	1.91E-02	229	-2.59E+00	1.02E-02	*	
ReplicateFragments:ROIROI2:YearYear 1	-5.56E-02	1.89E-02	229	-2.93E+00	3.67E-03	**	
ReplicateFibers:ROIROI3:YearYear 1	1.37E-02	1.91E-02	229	7.20E-01	4.72E-01		
ReplicateFragments:ROIROI3:YearYear 1	3.68E-03	1.89E-02	229	1.95E-01	8.46E-01		
ReplicateFibers:ROIROI2:YearYear 2	-9.27E-02	2.09E-02	236	-4.44E+00	1.38E-05	***	
ReplicateFragments:ROIROI2:YearYear 2	-5.90E-02	2.04E-02	235	-2.89E+00	4.17E-03	**	
ReplicateFibers:ROIROI3:YearYear 2	1.39E-02	2.10E-02	234	6.59E-01	5.10E-01		
ReplicateFragments:ROIROI3:YearYear 2	7.37E-03	1.99E-02	234	3.71E-01	7.11E-01		

Table S5.2: Post-hoc comparisons for connection probability showing pairwise differences across replicate, ROI, and years. Compairing between replicates within each ROI and year, and temporal differences across years within each replicate and ROI. Results include estimates, standard errors (SE), degrees of freedom (df), t-ratios, p-values, and significance levels, highlighting significant differences over time and between groups. Statistical significance is indicated as follows: p-value <0.001 ***, <0.01 **, <0.05 *, and <0.1 •.

**Connection Probability**								
**Post-hoc Comparisons**								
**Contrast**	**ROI**	**Year**	**Estimate**	**SE**	**df**	**t.ratio**	**p.value**	**Significance**
Control - Fibers	ROI1	Year 0	-6.80E-03	9.70E-03	236	-7.01E-01	7.63E-01	
Control - Fragments	ROI1	Year 0	-2.39E-03	9.32E-03	237	-2.57E-01	9.64E-01	
Fibers - Fragments	ROI1	Year 0	4.41E-03	9.77E-03	241	4.51E-01	8.94E-01	
Control - Fibers	ROI2	Year 0	-7.44E-02	9.70E-03	236	-7.67E+00	1.40E-12	***
Control - Fragments	ROI2	Year 0	-5.66E-02	9.77E-03	241	-5.79E+00	6.48E-08	***
Fibers - Fragments	ROI2	Year 0	1.78E-02	1.02E-02	246	1.74E+00	1.94E-01	
Control - Fibers	ROI3	Year 0	5.13E-04	9.52E-03	238	5.38E-02	9.98E-01	
Control - Fragments	ROI3	Year 0	8.90E-05	9.53E-03	238	9.34E-03	1.00E+00	
Fibers - Fragments	ROI3	Year 0	-4.24E-04	9.74E-03	241	-4.35E-02	9.99E-01	
Control - Fibers	ROI1	Year 1	5.55E-03	9.32E-03	237	5.96E-01	8.23E-01	
Control - Fragments	ROI1	Year 1	9.29E-04	9.46E-03	235	9.82E-02	9.95E-01	
Fibers - Fragments	ROI1	Year 1	-4.62E-03	9.53E-03	239	-4.85E-01	8.79E-01	
Control - Fibers	ROI2	Year 1	-1.24E-02	9.73E-03	240	-1.28E+00	4.09E-01	
Control - Fragments	ROI2	Year 1	2.31E-03	9.52E-03	238	2.43E-01	9.68E-01	
Fibers - Fragments	ROI2	Year 1	1.48E-02	9.53E-03	238	1.55E+00	2.70E-01	
Control - Fibers	ROI3	Year 1	-8.54E-04	9.78E-03	242	-8.73E-02	9.96E-01	
Control - Fragments	ROI3	Year 1	-2.71E-04	9.70E-03	237	-2.80E-02	1.00E+00	
Fibers - Fragments	ROI3	Year 1	5.83E-04	1.02E-02	242	5.74E-02	9.98E-01	
Control - Fibers	ROI1	Year 2	-1.68E-02	1.16E-02	253	-1.45E+00	3.19E-01	
Control - Fragments	ROI1	Year 2	-1.17E-02	1.06E-02	250	-1.10E+00	5.16E-01	
Fibers - Fragments	ROI1	Year 2	5.15E-03	1.26E-02	245	4.08E-01	9.12E-01	
Control - Fibers	ROI2	Year 2	8.34E-03	1.11E-02	257	7.54E-01	7.31E-01	
Control - Fragments	ROI2	Year 2	-6.83E-03	1.15E-02	260	-5.93E-01	8.24E-01	
Fibers - Fragments	ROI2	Year 2	-1.52E-02	1.21E-02	242	-1.25E+00	4.23E-01	
Control - Fibers	ROI3	Year 2	-2.34E-02	1.16E-02	253	-2.01E+00	1.12E-01	
Control - Fragments	ROI3	Year 2	-1.65E-02	1.06E-02	250	-1.56E+00	2.66E-01	
Fibers - Fragments	ROI3	Year 2	6.82E-03	1.26E-02	246	5.40E-01	8.52E-01	
**Contrast**	**Replicate**	**ROI**	**Estimate**	**SE**	**df**	**t.ratio**	**p.value**	**Significance**
Year 0 - Year 1	Control	ROI1	1.64E-02	1.02E-02	195	1.60E+00	2.49E-01	
Year 0 - Year 2	Control	ROI1	4.41E-02	1.01E-02	190	4.38E+00	5.81E-05	***
Year 1 - Year 2	Control	ROI1	2.78E-02	1.01E-02	189	2.76E+00	1.76E-02	*
Year 0 - Year 1	Fibers	ROI1	2.87E-02	1.06E-02	203	2.70E+00	2.03E-02	*
Year 0 - Year 2	Fibers	ROI1	3.41E-02	1.28E-02	242	2.66E+00	2.24E-02	*
Year 1 - Year 2	Fibers	ROI1	5.41E-03	1.25E-02	238	4.33E-01	9.02E-01	
Year 0 - Year 1	Fragments	ROI1	1.97E-02	1.04E-02	197	1.89E+00	1.44E-01	
Year 0 - Year 2	Fragments	ROI1	3.49E-02	1.16E-02	221	3.02E+00	8.01E-03	**
Year 1 - Year 2	Fragments	ROI1	1.52E-02	1.17E-02	225	1.29E+00	4.01E-01	
Year 0 - Year 1	Control	ROI2	4.86E-02	1.04E-02	200	4.66E+00	1.74E-05	***
Year 0 - Year 2	Control	ROI2	-5.33E-03	1.04E-02	200	-5.10E-01	8.66E-01	
Year 1 - Year 2	Control	ROI2	-5.39E-02	1.06E-02	203	-5.09E+00	2.43E-06	***
Year 0 - Year 1	Fibers	ROI2	1.11E-01	1.08E-02	209	1.02E+01	2.11E-14	***
Year 0 - Year 2	Fibers	ROI2	7.74E-02	1.19E-02	227	6.50E+00	1.53E-09	***
Year 1 - Year 2	Fibers	ROI2	-3.31E-02	1.17E-02	226	-2.82E+00	1.44E-02	*
Year 0 - Year 1	Fragments	ROI2	1.08E-01	1.06E-02	203	1.01E+01	3.95E-14	***
Year 0 - Year 2	Fragments	ROI2	4.45E-02	1.23E-02	235	3.61E+00	1.09E-03	**
Year 1 - Year 2	Fragments	ROI2	-6.31E-02	1.20E-02	229	-5.26E+00	9.80E-07	***
Year 0 - Year 1	Control	ROI3	1.39E-02	1.02E-02	195	1.36E+00	3.65E-01	
Year 0 - Year 2	Control	ROI3	5.54E-02	1.01E-02	190	5.50E+00	3.64E-07	***
Year 1 - Year 2	Control	ROI3	4.15E-02	1.01E-02	190	4.12E+00	1.69E-04	***
Year 0 - Year 1	Fibers	ROI3	1.25E-02	1.08E-02	207	1.16E+00	4.76E-01	
Year 0 - Year 2	Fibers	ROI3	3.15E-02	1.26E-02	239	2.50E+00	3.52E-02	*
Year 1 - Year 2	Fibers	ROI3	1.90E-02	1.28E-02	244	1.48E+00	3.03E-01	
Year 0 - Year 1	Fragments	ROI3	1.35E-02	1.08E-02	206	1.26E+00	4.21E-01	
Year 0 - Year 2	Fragments	ROI3	3.87E-02	1.17E-02	226	3.30E+00	3.21E-03	**
Year 1 - Year 2	Fragments	ROI3	2.52E-02	1.20E-02	231	2.11E+00	9.04E-02	.

Table S6.1: Summary of statistical analyses from a three-way ANOVA assessing the effects of replicate, ROI, year, and their interactions on biopore volume fraction. The table includes information on sums of squares, mean squares, degrees of freedom, F-values, and p-values for each main effect and interaction. Statistical significance is indicated as follows: p-value <0.001 ***, <0.01 **, <0.05 *, and <0.1 •.

**Biopore Volume Fraction**							
**ANOVA Results**							
	**Sum Sq**	**Mean Sq**	**NumDF**	**DenDF**	**F value**	**Pr(>F)**	**Significance**
Replicate	3.24E+00	1.62E+00	2	2.92E+02	1.53E+01	4.85E-07	***
ROI	3.53E+01	1.76E+01	2	2.77E+02	1.67E+02	2.76E-48	***
Year	2.11E+01	1.05E+01	2	2.96E+01	9.96E+01	7.21E-14	***
Replicate:ROI	7.67E-01	1.92E-01	4	2.77E+02	1.81E+00	1.27E-01	
Replicate:Year	1.52E+00	3.80E-01	4	2.92E+02	3.59E+00	7.10E-03	**
ROI:Year	9.15E+00	2.29E+00	4	2.77E+02	2.16E+01	1.53E-15	***
Replicate:ROI:Year	8.88E-01	1.11E-01	8	2.77E+02	1.05E+00	3.99E-01	
**Scaled Residuals**							
	**Min.**	**1st Qu.**	**Median**	**Mean**	**3rd Qu.**	**Max.**	
**Freq**	-3.23E+00	-6.52E-01	-6.22E-02	-1.11E-17	6.31E-01	3.68E+00	
**Random Effects**							
**grp**	**var1**	**vcov**	**sdcor**	**Std.Dev.**			
Block:ROI	(Intercept)	0	0	0			
Block:Year	(Intercept)	3.37E-02	1.84E-01	1.84E-01			
Block	(Intercept)	6.90E-03	8.31E-02	8.31E-02			
Residual		1.06E-01	3.25E-01	3.25E-01			
**Fixed Effects**							
	**Estimate**	**Std. Error**	**df**	**t value**	**Pr(>|t|)**	**Significance**	
(Intercept)	1.00E+00	9.88E-02	204	1.02E+01	7.13E-20	***	
ReplicateFibers	7.10E-02	1.21E-01	277	5.86E-01	5.58E-01		
ReplicateFragments	2.24E-01	1.21E-01	277	1.85E+00	6.60E-02	.	
ROIROI2	7.67E-01	1.19E-01	277	6.46E+00	4.76E-10	***	
ROIROI3	-3.20E-01	1.19E-01	277	-2.69E+00	7.48E-03	**	
YearYear 1	1.19E+00	1.36E-01	174	8.76E+00	1.73E-15	***	
YearYear 2	1.14E+00	1.36E-01	174	8.33E+00	2.33E-14	***	
ReplicateFibers:ROIROI2	2.66E-01	1.73E-01	277	1.54E+00	1.25E-01		
ReplicateFragments:ROIROI2	1.64E-01	1.71E-01	277	9.59E-01	3.38E-01		
ReplicateFibers:ROIROI3	5.75E-02	1.75E-01	277	3.29E-01	7.43E-01		
ReplicateFragments:ROIROI3	-4.75E-02	1.71E-01	277	-2.78E-01	7.82E-01		
ReplicateFibers:YearYear 1	9.64E-02	1.73E-01	278	5.57E-01	5.78E-01		
ReplicateFragments:YearYear 1	-2.47E-01	1.71E-01	278	-1.44E+00	1.50E-01		
ReplicateFibers:YearYear 2	7.27E-02	1.85E-01	285	3.93E-01	6.95E-01		
ReplicateFragments:YearYear 2	1.46E-01	1.85E-01	284	7.91E-01	4.29E-01		
ROIROI2:YearYear 1	-7.56E-01	1.68E-01	277	-4.50E+00	1.00E-05	***	
ROIROI3:YearYear 1	2.10E-01	1.68E-01	277	1.25E+00	2.12E-01		
ROIROI2:YearYear 2	-3.31E-01	1.68E-01	277	-1.97E+00	4.98E-02	*	
ROIROI3:YearYear 2	-1.23E-01	1.76E-01	279	-6.99E-01	4.85E-01		
ReplicateFibers:ROIROI2:YearYear 1	-2.07E-01	2.46E-01	277	-8.43E-01	4.00E-01		
ReplicateFragments:ROIROI2:YearYear 1	2.10E-01	2.42E-01	277	8.67E-01	3.87E-01		
ReplicateFibers:ROIROI3:YearYear 1	-1.92E-01	2.47E-01	277	-7.79E-01	4.37E-01		
ReplicateFragments:ROIROI3:YearYear 1	-1.60E-01	2.42E-01	277	-6.63E-01	5.08E-01		
ReplicateFibers:ROIROI2:YearYear 2	-3.13E-01	2.67E-01	278	-1.17E+00	2.42E-01		
ReplicateFragments:ROIROI2:YearYear 2	-1.07E-01	2.59E-01	277	-4.12E-01	6.80E-01		
ReplicateFibers:ROIROI3:YearYear 2	9.31E-02	2.70E-01	279	3.45E-01	7.30E-01		
ReplicateFragments:ROIROI3:YearYear 2	1.13E-01	2.64E-01	278	4.28E-01	6.69E-01		

Table S6.2: Post-hoc comparisons for biopore volume fraction showing pairwise differences across replicate, ROI, and years. Compairing between replicates within each ROI and year, and temporal differences across years within each replicate and ROI. Results include estimates, standard errors (SE), degrees of freedom (df), t-ratios, p-values, and significance levels, highlighting significant differences over time and between groups. Statistical significance is indicated as follows: p-value <0.001 ***, <0.01 **, <0.05 *, and <0.1 •.

Biopore Volume Fraction								
Post-hoc Comparisons								
Contrast	ROI	Year	Estimate	SE	df	t.ratio	p.value	Significance
Control - Fibers	ROI1	Year 0	-7.10E-02	1.21E-01	251	-5.86E-01	8.28E-01	
Control - Fragments	ROI1	Year 0	-2.24E-01	1.21E-01	250	-1.84E+00	1.57E-01	
Fibers - Fragments	ROI1	Year 0	-1.53E-01	1.24E-01	253	-1.23E+00	4.34E-01	
Control - Fibers	ROI2	Year 0	-3.37E-01	1.24E-01	253	-2.72E+00	1.90E-02	*
Control - Fragments	ROI2	Year 0	-3.87E-01	1.21E-01	251	-3.20E+00	4.44E-03	**
Fibers - Fragments	ROI2	Year 0	-5.04E-02	1.26E-01	256	-4.00E-01	9.16E-01	
Control - Fibers	ROI3	Year 0	-1.28E-01	1.27E-01	256	-1.01E+00	5.69E-01	
Control - Fragments	ROI3	Year 0	-1.76E-01	1.21E-01	251	-1.45E+00	3.15E-01	
Fibers - Fragments	ROI3	Year 0	-4.76E-02	1.29E-01	258	-3.68E-01	9.28E-01	
Control - Fibers	ROI1	Year 1	-1.67E-01	1.24E-01	253	-1.35E+00	3.68E-01	
Control - Fragments	ROI1	Year 1	2.38E-02	1.21E-01	251	1.97E-01	9.79E-01	
Fibers - Fragments	ROI1	Year 1	1.91E-01	1.26E-01	256	1.52E+00	2.85E-01	
Control - Fibers	ROI2	Year 1	-2.26E-01	1.24E-01	253	-1.83E+00	1.63E-01	
Control - Fragments	ROI2	Year 1	-3.50E-01	1.21E-01	251	-2.89E+00	1.18E-02	*
Fibers - Fragments	ROI2	Year 1	-1.23E-01	1.26E-01	256	-9.77E-01	5.92E-01	
Control - Fibers	ROI3	Year 1	-3.27E-02	1.24E-01	253	-2.64E-01	9.62E-01	
Control - Fragments	ROI3	Year 1	2.31E-01	1.21E-01	251	1.91E+00	1.38E-01	
Fibers - Fragments	ROI3	Year 1	2.64E-01	1.26E-01	256	2.09E+00	9.34E-02	.
Control - Fibers	ROI1	Year 2	-1.44E-01	1.41E-01	270	-1.02E+00	5.63E-01	
Control - Fragments	ROI1	Year 2	-3.70E-01	1.40E-01	270	-2.63E+00	2.41E-02	*
Fibers - Fragments	ROI1	Year 2	-2.26E-01	1.55E-01	256	-1.46E+00	3.10E-01	
Control - Fibers	ROI2	Year 2	-9.65E-02	1.53E-01	276	-6.29E-01	8.04E-01	
Control - Fragments	ROI2	Year 2	-4.27E-01	1.40E-01	270	-3.04E+00	7.26E-03	**
Fibers - Fragments	ROI2	Year 2	-3.31E-01	1.67E-01	265	-1.98E+00	1.18E-01	
Control - Fibers	ROI3	Year 2	-2.94E-01	1.59E-01	294	-1.85E+00	1.55E-01	
Control - Fragments	ROI3	Year 2	-4.36E-01	1.52E-01	284	-2.87E+00	1.23E-02	*
Fibers - Fragments	ROI3	Year 2	-1.41E-01	1.60E-01	262	-8.83E-01	6.52E-01	
Contrast	Replicate	ROI	Estimate	SE	df	t.ratio	p.value	Significance
Year 0 - Year 1	Control	ROI1	-1.19E+00	1.36E-01	165	-8.76E+00	1.12E-13	***
Year 0 - Year 2	Control	ROI1	-1.14E+00	1.36E-01	165	-8.33E+00	1.96E-13	***
Year 1 - Year 2	Control	ROI1	5.81E-02	1.36E-01	165	4.26E-01	9.05E-01	
Year 0 - Year 1	Fibers	ROI1	-1.29E+00	1.43E-01	180	-9.04E+00	3.33E-15	***
Year 0 - Year 2	Fibers	ROI1	-1.21E+00	1.57E-01	208	-7.68E+00	1.93E-12	***
Year 1 - Year 2	Fibers	ROI1	8.19E-02	1.59E-01	211	5.14E-01	8.65E-01	
Year 0 - Year 1	Fragments	ROI1	-9.47E-01	1.40E-01	174	-6.74E+00	6.60E-10	***
Year 0 - Year 2	Fragments	ROI1	-1.28E+00	1.57E-01	209	-8.15E+00	1.09E-13	***
Year 1 - Year 2	Fragments	ROI1	-3.36E-01	1.57E-01	209	-2.13E+00	8.58E-02	.
Year 0 - Year 1	Control	ROI2	-4.39E-01	1.36E-01	165	-3.22E+00	4.45E-03	**
Year 0 - Year 2	Control	ROI2	-8.05E-01	1.36E-01	165	-5.90E+00	5.88E-08	***
Year 1 - Year 2	Control	ROI2	-3.67E-01	1.36E-01	165	-2.69E+00	2.14E-02	*
Year 0 - Year 1	Fibers	ROI2	-3.28E-01	1.45E-01	186	-2.26E+00	6.40E-02	.
Year 0 - Year 2	Fibers	ROI2	-5.65E-01	1.71E-01	236	-3.30E+00	3.18E-03	**
Year 1 - Year 2	Fibers	ROI2	-2.37E-01	1.71E-01	233	-1.39E+00	3.50E-01	
Year 0 - Year 1	Fragments	ROI2	-4.01E-01	1.40E-01	174	-2.85E+00	1.34E-02	*
Year 0 - Year 2	Fragments	ROI2	-8.45E-01	1.57E-01	209	-5.37E+00	6.28E-07	***
Year 1 - Year 2	Fragments	ROI2	-4.45E-01	1.57E-01	209	-2.82E+00	1.44E-02	*
Year 0 - Year 1	Control	ROI3	-1.40E+00	1.36E-01	165	-1.03E+01	1.03E-13	***
Year 0 - Year 2	Control	ROI3	-1.01E+00	1.47E-01	191	-6.90E+00	2.23E-10	***
Year 1 - Year 2	Control	ROI3	3.91E-01	1.47E-01	191	2.67E+00	2.26E-02	*
Year 0 - Year 1	Fibers	ROI3	-1.31E+00	1.48E-01	192	-8.86E+00	0.00E+00	***
Year 0 - Year 2	Fibers	ROI3	-1.18E+00	1.67E-01	230	-7.06E+00	6.00E-11	***
Year 1 - Year 2	Fibers	ROI3	1.30E-01	1.65E-01	222	7.88E-01	7.11E-01	
Year 0 - Year 1	Fragments	ROI3	-9.97E-01	1.40E-01	174	-7.10E+00	9.25E-11	***
Year 0 - Year 2	Fragments	ROI3	-1.27E+00	1.57E-01	209	-8.08E+00	2.79E-13	***
Year 1 - Year 2	Fragments	ROI3	-2.76E-01	1.57E-01	209	-1.75E+00	1.89E-01	

Table S7.1: Summary of statistical analyses from a three-way ANOVA assessing the effects of replicate, ROI, year, and their interactions on biopore surface area. The table includes information on sums of squares, mean squares, degrees of freedom, F-values, and p-values for each main effect and interaction. Statistical significance is indicated as follows: p-value <0.001 ***, <0.01 **, <0.05 *, and <0.1 •.

**Biopore Surface Area**							
**ANOVA Results**							
	**Sum Sq**	**Mean Sq**	**NumDF**	**DenDF**	**F value**	**Pr(>F)**	**Significance**
Replicate	3.01E-04	1.50E-04	2	2.62E+02	1.61E+01	2.63E-07	***
ROI	2.29E-02	1.15E-02	2	3.07E+01	1.22E+03	5.48E-30	***
Year	7.78E-04	3.89E-04	2	2.88E+01	4.15E+01	3.29E-09	***
Replicate:ROI	7.12E-05	1.78E-05	4	2.57E+02	1.90E+00	1.11E-01	
Replicate:Year	8.25E-05	2.06E-05	4	2.63E+02	2.20E+00	6.93E-02	.
ROI:Year	9.91E-04	2.48E-04	4	2.56E+02	2.64E+01	2.28E-18	***
Replicate:ROI:Year	8.45E-05	1.06E-05	8	2.51E+02	1.13E+00	3.45E-01	
**Scaled Residuals**							
	**Min.**	**1st Qu.**	**Median**	**Mean**	**3rd Qu.**	**Max.**	
**Freq**	-4.39E+00	-4.70E-01	4.81E-02	1.10E-17	5.20E-01	2.75E+00	
**Random Effects**							
**grp**	**var1**	**vcov**	**sdcor**	**Std.Dev.**			
Block:ROI	(Intercept)	1.99E-07	4.46E-04	4.46E-04			
Block:Year	(Intercept)	9.81E-07	9.90E-04	9.90E-04			
Block	(Intercept)	6.41E-07	8.01E-04	8.01E-04			
Residual		9.37E-06	3.06E-03	3.06E-03			
**Fixed Effects**							
	**Estimate**	**Std. Error**	**df**	**t value**	**Pr(>|t|)**	**Significance**	
(Intercept)	2.28E-02	8.64E-04	255	2.64E+01	5.93E-75	***	
ReplicateFibers	1.84E-03	1.14E-03	245	1.61E+00	1.08E-01		
ReplicateFragments	4.83E-03	1.14E-03	245	4.24E+00	3.21E-05	***	
ROIROI2	-1.60E-02	1.15E-03	262	-1.39E+01	2.94E-33	***	
ROIROI3	-1.34E-02	1.13E-03	260	-1.19E+01	2.78E-26	***	
YearYear 1	8.65E-03	1.22E-03	243	7.10E+00	1.34E-11	***	
YearYear 2	3.91E-03	1.17E-03	235	3.33E+00	1.01E-03	**	
ReplicateFibers:ROIROI2	-7.07E-04	1.64E-03	247	-4.31E-01	6.67E-01		
ReplicateFragments:ROIROI2	-3.55E-03	1.62E-03	246	-2.19E+00	2.96E-02	*	
ReplicateFibers:ROIROI3	1.78E-04	1.65E-03	247	1.08E-01	9.14E-01		
ReplicateFragments:ROIROI3	-2.64E-03	1.61E-03	245	-1.64E+00	1.02E-01		
ReplicateFibers:YearYear 1	9.20E-04	1.64E-03	247	5.60E-01	5.76E-01		
ReplicateFragments:YearYear 1	-3.82E-03	1.64E-03	247	-2.32E+00	2.10E-02	*	
ReplicateFibers:YearYear 2	1.71E-03	1.77E-03	258	9.66E-01	3.35E-01		
ReplicateFragments:YearYear 2	3.90E-04	1.77E-03	258	2.20E-01	8.26E-01		
ROIROI2:YearYear 1	-9.47E-03	1.64E-03	247	-5.77E+00	2.39E-08	***	
ROIROI3:YearYear 1	6.67E-04	1.61E-03	245	4.13E-01	6.80E-01		
ROIROI2:YearYear 2	-4.72E-03	1.60E-03	244	-2.96E+00	3.41E-03	**	
ROIROI3:YearYear 2	-5.41E-05	1.60E-03	244	-3.39E-02	9.73E-01		
ReplicateFibers:ROIROI2:YearYear 1	-1.58E-03	2.36E-03	247	-6.69E-01	5.04E-01		
ReplicateFragments:ROIROI2:YearYear 1	4.10E-03	2.43E-03	252	1.68E+00	9.34E-02	.	
ReplicateFibers:ROIROI3:YearYear 1	-1.85E-03	2.34E-03	246	-7.90E-01	4.30E-01		
ReplicateFragments:ROIROI3:YearYear 1	6.84E-04	2.31E-03	245	2.96E-01	7.67E-01		
ReplicateFibers:ROIROI2:YearYear 2	-1.95E-03	2.51E-03	254	-7.75E-01	4.39E-01		
ReplicateFragments:ROIROI2:YearYear 2	-7.97E-04	2.47E-03	252	-3.22E-01	7.48E-01		
ReplicateFibers:ROIROI3:YearYear 2	-1.76E-03	2.53E-03	254	-6.94E-01	4.88E-01		
ReplicateFragments:ROIROI3:YearYear 2	1.27E-03	2.47E-03	251	5.14E-01	6.08E-01		

Table S7.2: Post-hoc comparisons for biopore surface area showing pairwise differences across replicate, ROI, and years. Compairing between replicates within each ROI and year, and temporal differences across years within each replicate and ROI. Results include estimates, standard errors (SE), degrees of freedom (df), t-ratios, p-values, and significance levels, highlighting significant differences over time and between groups. Statistical significance is indicated as follows: p-value <0.001 ***, <0.01 **, <0.05 *, and <0.1 •.

**Biopore Surface Area**								
**Post-hoc Comparisons**								
**Contrast**	**ROI**	**Year**	**Estimate**	**SE**	**df**	**t.ratio**	**p.value**	**Significance**
Control - Fibers	ROI1	Year 0	-1.84E-03	1.14E-03	242	-1.61E+00	2.43E-01	
Control - Fragments	ROI1	Year 0	-4.83E-03	1.14E-03	242	-4.24E+00	9.59E-05	***
Fibers - Fragments	ROI1	Year 0	-2.99E-03	1.16E-03	245	-2.57E+00	2.85E-02	*
Control - Fibers	ROI2	Year 0	-1.13E-03	1.19E-03	248	-9.51E-01	6.08E-01	
Control - Fragments	ROI2	Year 0	-1.27E-03	1.16E-03	245	-1.10E+00	5.17E-01	
Fibers - Fragments	ROI2	Year 0	-1.46E-04	1.19E-03	248	-1.23E-01	9.92E-01	
Control - Fibers	ROI3	Year 0	-2.01E-03	1.19E-03	248	-1.69E+00	2.12E-01	
Control - Fragments	ROI3	Year 0	-2.19E-03	1.14E-03	242	-1.92E+00	1.35E-01	
Fibers - Fragments	ROI3	Year 0	-1.75E-04	1.21E-03	251	-1.44E-01	9.89E-01	
Control - Fibers	ROI1	Year 1	-2.76E-03	1.19E-03	248	-2.32E+00	5.45E-02	.
Control - Fragments	ROI1	Year 1	-1.01E-03	1.19E-03	248	-8.53E-01	6.70E-01	
Fibers - Fragments	ROI1	Year 1	1.74E-03	1.16E-03	245	1.50E+00	2.92E-01	
Control - Fibers	ROI2	Year 1	-4.73E-04	1.22E-03	252	-3.89E-01	9.20E-01	
Control - Fragments	ROI2	Year 1	-1.55E-03	1.38E-03	266	-1.12E+00	5.01E-01	
Fibers - Fragments	ROI2	Year 1	-1.08E-03	1.43E-03	268	-7.57E-01	7.29E-01	
Control - Fibers	ROI3	Year 1	-1.09E-03	1.16E-03	245	-9.34E-01	6.19E-01	
Control - Fragments	ROI3	Year 1	9.44E-04	1.16E-03	245	8.10E-01	6.97E-01	
Fibers - Fragments	ROI3	Year 1	2.03E-03	1.21E-03	252	1.68E+00	2.16E-01	
Control - Fibers	ROI1	Year 2	-3.55E-03	1.37E-03	266	-2.60E+00	2.68E-02	*
Control - Fragments	ROI1	Year 2	-5.22E-03	1.37E-03	265	-3.82E+00	4.93E-04	***
Fibers - Fragments	ROI1	Year 2	-1.67E-03	1.55E-03	258	-1.07E+00	5.31E-01	
Control - Fibers	ROI2	Year 2	-8.94E-04	1.37E-03	266	-6.54E-01	7.90E-01	
Control - Fragments	ROI2	Year 2	-8.67E-04	1.31E-03	263	-6.60E-01	7.87E-01	
Fibers - Fragments	ROI2	Year 2	2.71E-05	1.50E-03	254	1.80E-02	1.00E+00	
Control - Fibers	ROI3	Year 2	-1.97E-03	1.39E-03	272	-1.42E+00	3.33E-01	
Control - Fragments	ROI3	Year 2	-3.85E-03	1.33E-03	261	-2.90E+00	1.13E-02	*
Fibers - Fragments	ROI3	Year 2	-1.88E-03	1.50E-03	254	-1.25E+00	4.26E-01	
**Contrast**	**Replicate**	**ROI**	**Estimate**	**SE**	**df**	**t.ratio**	**p.value**	**Significance**
Year 0 - Year 1	Control	ROI1	-8.65E-03	1.22E-03	240	-7.10E+00	4.26E-11	***
Year 0 - Year 2	Control	ROI1	-3.91E-03	1.17E-03	232	-3.33E+00	2.90E-03	**
Year 1 - Year 2	Control	ROI1	4.74E-03	1.22E-03	240	3.89E+00	3.80E-04	***
Year 0 - Year 1	Fibers	ROI1	-9.57E-03	1.21E-03	236	-7.88E+00	3.07E-13	***
Year 0 - Year 2	Fibers	ROI1	-5.62E-03	1.43E-03	262	-3.93E+00	3.17E-04	***
Year 1 - Year 2	Fibers	ROI1	3.95E-03	1.43E-03	262	2.76E+00	1.69E-02	*
Year 0 - Year 1	Fragments	ROI1	-4.84E-03	1.21E-03	236	-3.98E+00	2.66E-04	***
Year 0 - Year 2	Fragments	ROI1	-4.30E-03	1.43E-03	262	-3.01E+00	8.13E-03	**
Year 1 - Year 2	Fragments	ROI1	5.39E-04	1.43E-03	262	3.77E-01	9.25E-01	
Year 0 - Year 1	Control	ROI2	8.12E-04	1.22E-03	240	6.67E-01	7.83E-01	
Year 0 - Year 2	Control	ROI2	8.08E-04	1.20E-03	236	6.76E-01	7.78E-01	
Year 1 - Year 2	Control	ROI2	-3.23E-06	1.20E-03	236	-2.70E-03	1.00E+00	
Year 0 - Year 1	Fibers	ROI2	1.47E-03	1.29E-03	249	1.14E+00	4.91E-01	
Year 0 - Year 2	Fibers	ROI2	1.04E-03	1.45E-03	266	7.19E-01	7.53E-01	
Year 1 - Year 2	Fibers	ROI2	-4.24E-04	1.47E-03	261	-2.89E-01	9.55E-01	
Year 0 - Year 1	Fragments	ROI2	5.31E-04	1.43E-03	266	3.72E-01	9.26E-01	
Year 0 - Year 2	Fragments	ROI2	1.22E-03	1.38E-03	256	8.82E-01	6.52E-01	
Year 1 - Year 2	Fragments	ROI2	6.85E-04	1.57E-03	280	4.36E-01	9.01E-01	
Year 0 - Year 1	Control	ROI3	-9.32E-03	1.17E-03	232	-7.94E+00	3.39E-13	***
Year 0 - Year 2	Control	ROI3	-3.86E-03	1.20E-03	236	-3.22E+00	4.10E-03	**
Year 1 - Year 2	Control	ROI3	5.47E-03	1.20E-03	236	4.57E+00	2.34E-05	***
Year 0 - Year 1	Fibers	ROI3	-8.40E-03	1.29E-03	249	-6.52E+00	1.16E-09	***
Year 0 - Year 2	Fibers	ROI3	-3.81E-03	1.48E-03	270	-2.59E+00	2.76E-02	*
Year 1 - Year 2	Fibers	ROI3	4.58E-03	1.45E-03	261	3.16E+00	4.97E-03	**
Year 0 - Year 1	Fragments	ROI3	-6.19E-03	1.24E-03	240	-5.00E+00	3.33E-06	***
Year 0 - Year 2	Fragments	ROI3	-5.52E-03	1.38E-03	256	-4.00E+00	2.43E-04	***
Year 1 - Year 2	Fragments	ROI3	6.73E-04	1.40E-03	260	4.80E-01	8.81E-01	

Table S8.1: Summary of statistical analyses from a three-way ANOVA assessing the effects of replicate, ROI, year, and their interactions on POM volume fraction. The table includes information on sums of squares, mean squares, degrees of freedom, F-values, and p-values for each main effect and interaction. Statistical significance is indicated as follows: p-value <0.001 ***, <0.01 **, <0.05 *, and <0.1 •.

**POM Volume Fraction**							
**ANOVA Results**							
	**Sum Sq**	**Mean Sq**	**NumDF**	**DenDF**	**F value**	**Pr(>F)**	**Significance**
Replicate	1.28E+00	6.42E-01	2	2.61E+02	3.04E+00	4.93E-02	*
ROI	1.19E+01	5.96E+00	2	2.81E+01	2.83E+01	1.86E-07	***
Year	8.25E+00	4.13E+00	2	2.71E+01	1.96E+01	5.52E-06	***
Replicate:ROI	4.39E-01	1.10E-01	4	2.57E+02	5.20E-01	7.21E-01	
Replicate:Year	3.77E-01	9.42E-02	4	2.63E+02	4.47E-01	7.75E-01	
ROI:Year	1.67E+01	4.17E+00	4	2.56E+02	1.98E+01	3.23E-14	***
Replicate:ROI:Year	2.29E-01	2.86E-02	8	2.53E+02	1.36E-01	9.98E-01	
**Scaled Residuals**							
	**Min.**	**1st Qu.**	**Median**	**Mean**	**3rd Qu.**	**Max.**	
**Freq**	-3.16E+00	-5.55E-01	-6.89E-02	-2.45E-17	4.88E-01	3.94E+00	
**Random Effects**							
**grp**	**var1**	**vcov**	**sdcor**	**Std.Dev.**			
Block:ROI	(Intercept)	4.46E-02	2.11E-01	2.11E-01			
Block:Year	(Intercept)	5.32E-02	2.31E-01	2.31E-01			
Block	(Intercept)	4.30E-02	2.07E-01	2.07E-01			
Residual		2.11E-01	4.59E-01	4.59E-01			
**Fixed Effects**							
	**Estimate**	**Std. Error**	**df**	**t value**	**Pr(>|t|)**	**Significance**	
(Intercept)	1.42E+00	1.57E-01	162	9.05E+00	4.12E-16	***	
ReplicateFibers	-7.52E-03	1.79E-01	256	-4.20E-02	9.67E-01		
ReplicateFragments	-8.29E-02	1.74E-01	250	-4.78E-01	6.33E-01		
ROIROI2	-9.71E-01	1.88E-01	205	-5.17E+00	5.55E-07	***	
ROIROI3	2.24E-01	1.90E-01	208	1.18E+00	2.41E-01		
YearYear 1	2.06E-01	1.91E-01	185	1.08E+00	2.81E-01		
YearYear 2	-5.72E-01	1.91E-01	185	-3.00E+00	3.10E-03	**	
ReplicateFibers:ROIROI2	-2.49E-01	2.53E-01	255	-9.84E-01	3.26E-01		
ReplicateFragments:ROIROI2	-1.88E-01	2.50E-01	252	-7.55E-01	4.51E-01		
ReplicateFibers:ROIROI3	-4.21E-02	2.51E-01	254	-1.68E-01	8.67E-01		
ReplicateFragments:ROIROI3	4.67E-02	2.45E-01	250	1.90E-01	8.49E-01		
ReplicateFibers:YearYear 1	1.11E-01	2.47E-01	253	4.50E-01	6.53E-01		
ReplicateFragments:YearYear 1	-5.49E-02	2.44E-01	251	-2.25E-01	8.22E-01		
ReplicateFibers:YearYear 2	8.51E-02	2.66E-01	260	3.19E-01	7.50E-01		
ReplicateFragments:YearYear 2	-2.34E-02	2.70E-01	260	-8.67E-02	9.31E-01		
ROIROI2:YearYear 1	4.47E-01	2.40E-01	251	1.86E+00	6.36E-02	.	
ROIROI3:YearYear 1	4.31E-02	2.42E-01	251	1.78E-01	8.59E-01		
ROIROI2:YearYear 2	1.07E+00	2.40E-01	251	4.47E+00	1.20E-05	***	
ROIROI3:YearYear 2	-2.71E-01	2.42E-01	251	-1.12E+00	2.63E-01		
ReplicateFibers:ROIROI2:YearYear 1	1.78E-01	3.51E-01	252	5.07E-01	6.13E-01		
ReplicateFragments:ROIROI2:YearYear 1	1.49E-01	3.47E-01	251	4.30E-01	6.67E-01		
ReplicateFibers:ROIROI3:YearYear 1	-1.00E-01	3.48E-01	251	-2.89E-01	7.73E-01		
ReplicateFragments:ROIROI3:YearYear 1	-6.15E-02	3.44E-01	250	-1.79E-01	8.58E-01		
ReplicateFibers:ROIROI2:YearYear 2	1.05E-01	3.78E-01	254	2.78E-01	7.81E-01		
ReplicateFragments:ROIROI2:YearYear 2	4.01E-04	3.77E-01	256	1.06E-03	9.99E-01		
ReplicateFibers:ROIROI3:YearYear 2	9.44E-02	3.72E-01	252	2.53E-01	8.00E-01		
ReplicateFragments:ROIROI3:YearYear 2	4.18E-02	3.78E-01	254	1.11E-01	9.12E-01		

Table S8.2: Post-hoc comparisons for POM volume fraction showing pairwise differences across replicate, ROI, and years. Compairing between replicates within each ROI and year, and temporal differences across years within each replicate and ROI. Results include estimates, standard errors (SE), degrees of freedom (df), t-ratios, p-values, and significance levels, highlighting significant differences over time and between groups. Statistical significance is indicated as follows: p-value <0.001 ***, <0.01 **, <0.05 *, and <0.1 •.

**POM Volume Fraction**								
**Post-hoc Comparisons**								
**Contrast**	**ROI**	**Year**	**Estimate**	**SE**	**df**	**t.ratio**	**p.value**	**Significance**
Control - Fibers	ROI1	Year 0	7.52E-03	1.79E-01	255	4.20E-02	9.99E-01	
Control - Fragments	ROI1	Year 0	8.29E-02	1.74E-01	249	4.78E-01	8.82E-01	
Fibers - Fragments	ROI1	Year 0	7.54E-02	1.79E-01	255	4.21E-01	9.07E-01	
Control - Fibers	ROI2	Year 0	2.56E-01	1.80E-01	253	1.43E+00	3.28E-01	
Control - Fragments	ROI2	Year 0	2.71E-01	1.80E-01	253	1.51E+00	2.88E-01	
Fibers - Fragments	ROI2	Year 0	1.51E-02	1.92E-01	260	7.85E-02	9.97E-01	
Control - Fibers	ROI3	Year 0	4.97E-02	1.79E-01	255	2.77E-01	9.58E-01	
Control - Fragments	ROI3	Year 0	3.62E-02	1.74E-01	249	2.09E-01	9.76E-01	
Fibers - Fragments	ROI3	Year 0	-1.34E-02	1.79E-01	255	-7.50E-02	9.97E-01	
Control - Fibers	ROI1	Year 1	-1.04E-01	1.71E-01	250	-6.06E-01	8.17E-01	
Control - Fragments	ROI1	Year 1	1.38E-01	1.71E-01	250	8.05E-01	7.00E-01	
Fibers - Fragments	ROI1	Year 1	2.42E-01	1.75E-01	252	1.38E+00	3.52E-01	
Control - Fibers	ROI2	Year 1	-3.30E-02	1.75E-01	252	-1.88E-01	9.81E-01	
Control - Fragments	ROI2	Year 1	1.77E-01	1.71E-01	250	1.03E+00	5.56E-01	
Fibers - Fragments	ROI2	Year 1	2.10E-01	1.79E-01	254	1.17E+00	4.69E-01	
Control - Fibers	ROI3	Year 1	3.89E-02	1.71E-01	250	2.27E-01	9.72E-01	
Control - Fragments	ROI3	Year 1	1.53E-01	1.71E-01	250	8.92E-01	6.46E-01	
Fibers - Fragments	ROI3	Year 1	1.14E-01	1.75E-01	252	6.51E-01	7.92E-01	
Control - Fibers	ROI1	Year 2	-7.76E-02	1.99E-01	265	-3.89E-01	9.20E-01	
Control - Fragments	ROI1	Year 2	1.06E-01	2.08E-01	266	5.12E-01	8.66E-01	
Fibers - Fragments	ROI1	Year 2	1.84E-01	2.27E-01	257	8.10E-01	6.97E-01	
Control - Fibers	ROI2	Year 2	6.60E-02	2.08E-01	266	3.18E-01	9.46E-01	
Control - Fragments	ROI2	Year 2	2.94E-01	1.99E-01	265	1.48E+00	3.03E-01	
Fibers - Fragments	ROI2	Year 2	2.28E-01	2.27E-01	257	1.01E+00	5.73E-01	
Control - Fibers	ROI3	Year 2	-1.30E-01	1.99E-01	265	-6.51E-01	7.92E-01	
Control - Fragments	ROI3	Year 2	1.79E-02	2.08E-01	266	8.60E-02	9.96E-01	
Fibers - Fragments	ROI3	Year 2	1.48E-01	2.27E-01	257	6.51E-01	7.92E-01	
**Contrast**	**Replicate**	**ROI**	**Estimate**	**SE**	**df**	**t.ratio**	**p.value**	**Significance**
Year 0 - Year 1	Control	ROI1	-2.06E-01	1.91E-01	190	-1.08E+00	5.27E-01	
Year 0 - Year 2	Control	ROI1	5.72E-01	1.91E-01	190	3.00E+00	8.66E-03	**
Year 1 - Year 2	Control	ROI1	7.79E-01	1.88E-01	184	4.15E+00	1.50E-04	***
Year 0 - Year 1	Fibers	ROI1	-3.18E-01	1.97E-01	199	-1.61E+00	2.43E-01	
Year 0 - Year 2	Fibers	ROI1	4.87E-01	2.21E-01	226	2.20E+00	7.33E-02	.
Year 1 - Year 2	Fibers	ROI1	8.05E-01	2.19E-01	224	3.68E+00	8.54E-04	***
Year 0 - Year 1	Fragments	ROI1	-1.52E-01	1.94E-01	193	-7.83E-01	7.14E-01	
Year 0 - Year 2	Fragments	ROI1	5.96E-01	2.27E-01	233	2.63E+00	2.47E-02	*
Year 1 - Year 2	Fragments	ROI1	7.47E-01	2.26E-01	233	3.30E+00	3.21E-03	**
Year 0 - Year 1	Control	ROI2	-6.53E-01	1.88E-01	184	-3.48E+00	1.80E-03	**
Year 0 - Year 2	Control	ROI2	-4.99E-01	1.88E-01	184	-2.66E+00	2.32E-02	*
Year 1 - Year 2	Control	ROI2	1.54E-01	1.88E-01	184	8.22E-01	6.90E-01	
Year 0 - Year 1	Fibers	ROI2	-9.42E-01	2.04E-01	211	-4.61E+00	2.08E-05	***
Year 0 - Year 2	Fibers	ROI2	-6.89E-01	2.33E-01	240	-2.96E+00	9.44E-03	**
Year 1 - Year 2	Fibers	ROI2	2.53E-01	2.29E-01	236	1.11E+00	5.12E-01	
Year 0 - Year 1	Fragments	ROI2	-7.47E-01	2.01E-01	205	-3.72E+00	7.54E-04	***
Year 0 - Year 2	Fragments	ROI2	-4.76E-01	2.26E-01	234	-2.10E+00	9.11E-02	.
Year 1 - Year 2	Fragments	ROI2	2.72E-01	2.19E-01	224	1.24E+00	4.30E-01	
Year 0 - Year 1	Control	ROI3	-2.49E-01	1.91E-01	190	-1.31E+00	3.93E-01	
Year 0 - Year 2	Control	ROI3	8.43E-01	1.91E-01	190	4.42E+00	5.01E-05	***
Year 1 - Year 2	Control	ROI3	1.09E+00	1.88E-01	184	5.82E+00	7.55E-08	***
Year 0 - Year 1	Fibers	ROI3	-2.60E-01	1.97E-01	199	-1.32E+00	3.85E-01	
Year 0 - Year 2	Fibers	ROI3	6.64E-01	2.21E-01	226	3.00E+00	8.46E-03	**
Year 1 - Year 2	Fibers	ROI3	9.24E-01	2.19E-01	224	4.22E+00	1.03E-04	***
Year 0 - Year 1	Fragments	ROI3	-1.33E-01	1.94E-01	193	-6.88E-01	7.71E-01	
Year 0 - Year 2	Fragments	ROI3	8.25E-01	2.27E-01	233	3.64E+00	9.74E-04	***
Year 1 - Year 2	Fragments	ROI3	9.58E-01	2.26E-01	233	4.23E+00	9.95E-05	***

Table S9.1: Summary of statistical analyses from a three-way ANOVA assessing the effects of replicate, ROI, year, and their interactions on POM surface area. The table includes information on sums of squares, mean squares, degrees of freedom, F-values, and p-values for each main effect and interaction. Statistical significance is indicated as follows: p-value <0.001 ***, <0.01 **, <0.05 *, and <0.1 •.

Macropore Surface Area							
**ANOVA Results**							
	**Sum Sq**	**Mean Sq**	**NumDF**	**DenDF**	**F value**	**Pr(>F)**	**Significance**
Replicate	2.43E-03	1.22E-03	2	2.61E+02	3.77E+00	2.43E-02	*
ROI	2.02E+00	1.01E+00	2	3.02E+01	3.14E+03	1.03E-35	***
Year	3.14E-01	1.57E-01	2	2.57E+01	4.87E+02	3.72E-21	***
Replicate:ROI	5.00E-04	1.25E-04	4	2.57E+02	3.87E-01	8.18E-01	
Replicate:Year	1.09E-02	2.72E-03	4	2.63E+02	8.42E+00	2.10E-06	***
ROI:Year	2.81E-01	7.02E-02	4	2.56E+02	2.18E+02	4.18E-81	***
Replicate:ROI:Year	7.88E-03	9.86E-04	8	2.51E+02	3.06E+00	2.67E-03	**
**Scaled Residuals**							
	**Min.**	**1st Qu.**	**Median**	**Mean**	**3rd Qu.**	**Max.**	
**Freq**	-3.26E+00	-5.58E-01	-2.52E-02	-1.98E-17	5.52E-01	3.72E+00	
**Random Effects**							
**grp**	**var1**	**vcov**	**sdcor**	**Std.Dev.**			
Block:ROI	(Intercept)	3.42E-05	5.85E-03	5.85E-03			
Block:Year	(Intercept)	5.49E-05	7.41E-03	7.41E-03			
Block	(Intercept)	7.07E-05	8.41E-03	8.41E-03			
Residual		3.23E-04	1.80E-02	1.80E-02			
**Fixed Effects**							
	**Estimate**	**Std. Error**	**df**	**t value**	**Pr(>|t|)**	**Significance**	
(Intercept)	4.05E-01	5.67E-03	159	7.14E+01	1.12E-122	***	
ReplicateFibers	1.97E-02	6.69E-03	249	2.94E+00	3.57E-03	**	
ReplicateFragments	-3.59E-03	6.69E-03	249	-5.36E-01	5.92E-01		
ROIROI2	-3.55E-01	6.90E-03	239	-5.15E+01	2.00E-131	***	
ROIROI3	-1.34E-01	6.90E-03	239	-1.94E+01	7.08E-51	***	
YearYear 1	-1.25E-01	7.09E-03	207	-1.76E+01	6.04E-43	***	
YearYear 2	-1.99E-01	7.09E-03	207	-2.81E+01	1.38E-72	***	
ReplicateFibers:ROIROI2	-7.42E-03	9.45E-03	248	-7.85E-01	4.33E-01		
ReplicateFragments:ROIROI2	1.45E-02	9.68E-03	250	1.50E+00	1.35E-01		
ReplicateFibers:ROIROI3	-1.98E-02	9.56E-03	249	-2.08E+00	3.89E-02	*	
ReplicateFragments:ROIROI3	-1.36E-02	9.45E-03	248	-1.44E+00	1.52E-01		
ReplicateFibers:YearYear 1	-3.92E-02	9.68E-03	250	-4.05E+00	6.85E-05	***	
ReplicateFragments:YearYear 1	2.72E-03	9.45E-03	248	2.88E-01	7.73E-01		
ReplicateFibers:YearYear 2	6.23E-03	1.05E-02	260	5.95E-01	5.52E-01		
ReplicateFragments:YearYear 2	3.35E-02	1.02E-02	259	3.28E+00	1.18E-03	**	
ROIROI2:YearYear 1	1.14E-01	9.28E-03	247	1.23E+01	2.08E-27	***	
ROIROI3:YearYear 1	3.50E-02	9.28E-03	247	3.78E+00	1.98E-04	***	
ROIROI2:YearYear 2	1.86E-01	9.38E-03	249	1.98E+01	3.61E-53	***	
ROIROI3:YearYear 2	4.74E-02	9.37E-03	248	5.06E+00	8.23E-07	***	
ReplicateFibers:ROIROI2:YearYear 1	2.88E-02	1.37E-02	249	2.11E+00	3.60E-02	*	
ReplicateFragments:ROIROI2:YearYear 1	-1.28E-02	1.35E-02	248	-9.50E-01	3.43E-01		
ReplicateFibers:ROIROI3:YearYear 1	3.24E-02	1.36E-02	249	2.38E+00	1.80E-02	*	
ReplicateFragments:ROIROI3:YearYear 1	1.59E-02	1.34E-02	247	1.18E+00	2.39E-01		
ReplicateFibers:ROIROI2:YearYear 2	-1.37E-02	1.47E-02	253	-9.32E-01	3.52E-01		
ReplicateFragments:ROIROI2:YearYear 2	-4.11E-02	1.45E-02	252	-2.83E+00	5.10E-03	**	
ReplicateFibers:ROIROI3:YearYear 2	7.26E-03	1.50E-02	254	4.84E-01	6.29E-01		
ReplicateFragments:ROIROI3:YearYear 2	9.69E-03	1.44E-02	252	6.73E-01	5.02E-01		

Table S9.2: Post-hoc comparisons for POM surface area showing pairwise differences across replicate, ROI, and years. Compairing between replicates within each ROI and year, and temporal differences across years within each replicate and ROI. Results include estimates, standard errors (SE), degrees of freedom (df), t-ratios, p-values, and significance levels, highlighting significant differences over time and between groups. Statistical significance is indicated as follows: p-value <0.001 ***, <0.01 **, <0.05 *, and <0.1 •.

POM Surface Area								
Post-hoc Comparisons								
Contrast	ROI	Year	Estimate	SE	df	t.ratio	p.value	Significance
Control - Fibers	ROI1	Year 0	-1.47E-03	2.92E-03	251	-5.02E-01	8.71E-01	
Control - Fragments	ROI1	Year 0	3.60E-03	2.92E-03	251	1.23E+00	4.35E-01	
Fibers - Fragments	ROI1	Year 0	5.07E-03	2.98E-03	253	1.70E+00	2.08E-01	
Control - Fibers	ROI2	Year 0	7.39E-04	2.99E-03	253	2.47E-01	9.67E-01	
Control - Fragments	ROI2	Year 0	6.64E-04	2.99E-03	252	2.22E-01	9.73E-01	
Fibers - Fragments	ROI2	Year 0	-7.50E-05	3.12E-03	256	-2.40E-02	1.00E+00	
Control - Fibers	ROI3	Year 0	-1.26E-03	2.92E-03	251	-4.30E-01	9.03E-01	
Control - Fragments	ROI3	Year 0	2.22E-03	2.92E-03	251	7.60E-01	7.28E-01	
Fibers - Fragments	ROI3	Year 0	3.48E-03	2.98E-03	253	1.17E+00	4.75E-01	
Control - Fibers	ROI1	Year 1	9.79E-04	2.99E-03	252	3.28E-01	9.43E-01	
Control - Fragments	ROI1	Year 1	2.33E-03	2.92E-03	251	7.96E-01	7.06E-01	
Fibers - Fragments	ROI1	Year 1	1.35E-03	3.05E-03	254	4.42E-01	8.98E-01	
Control - Fibers	ROI2	Year 1	-2.64E-04	2.92E-03	251	-9.05E-02	9.95E-01	
Control - Fragments	ROI2	Year 1	7.33E-04	2.99E-03	253	2.45E-01	9.67E-01	
Fibers - Fragments	ROI2	Year 1	9.97E-04	3.05E-03	254	3.27E-01	9.43E-01	
Control - Fibers	ROI3	Year 1	8.50E-04	2.92E-03	251	2.91E-01	9.54E-01	
Control - Fragments	ROI3	Year 1	1.23E-03	2.92E-03	251	4.21E-01	9.07E-01	
Fibers - Fragments	ROI3	Year 1	3.80E-04	2.98E-03	253	1.28E-01	9.91E-01	
Control - Fibers	ROI1	Year 2	-8.19E-04	3.58E-03	265	-2.28E-01	9.72E-01	
Control - Fragments	ROI1	Year 2	1.68E-03	3.76E-03	266	4.48E-01	8.95E-01	
Fibers - Fragments	ROI1	Year 2	2.50E-03	4.13E-03	257	6.06E-01	8.17E-01	
Control - Fibers	ROI2	Year 2	5.77E-04	3.41E-03	265	1.70E-01	9.84E-01	
Control - Fragments	ROI2	Year 2	1.97E-03	3.55E-03	266	5.55E-01	8.44E-01	
Fibers - Fragments	ROI2	Year 2	1.39E-03	3.87E-03	257	3.60E-01	9.31E-01	
Control - Fibers	ROI3	Year 2	-8.94E-04	3.56E-03	267	-2.51E-01	9.66E-01	
Control - Fragments	ROI3	Year 2	-1.13E-03	3.55E-03	267	-3.17E-01	9.46E-01	
Fibers - Fragments	ROI3	Year 2	-2.32E-04	3.96E-03	255	-5.86E-02	9.98E-01	
Contrast	Replicate	ROI	Estimate	SE	df	t.ratio	p.value	Significance
Year 0 - Year 1	Control	ROI1	-4.49E-03	3.21E-03	181	-1.40E+00	3.44E-01	
Year 0 - Year 2	Control	ROI1	1.43E-02	3.27E-03	185	4.35E+00	6.53E-05	***
Year 1 - Year 2	Control	ROI1	1.88E-02	3.27E-03	185	5.73E+00	1.23E-07	***
Year 0 - Year 1	Fibers	ROI1	-2.05E-03	3.37E-03	195	-6.07E-01	8.17E-01	
Year 0 - Year 2	Fibers	ROI1	1.49E-02	3.89E-03	229	3.83E+00	4.78E-04	***
Year 1 - Year 2	Fibers	ROI1	1.70E-02	3.95E-03	234	4.30E+00	7.54E-05	***
Year 0 - Year 1	Fragments	ROI1	-5.77E-03	3.31E-03	190	-1.74E+00	1.93E-01	
Year 0 - Year 2	Fragments	ROI1	1.23E-02	4.05E-03	239	3.05E+00	7.19E-03	**
Year 1 - Year 2	Fragments	ROI1	1.81E-02	4.05E-03	239	4.47E+00	3.55E-05	***
Year 0 - Year 1	Control	ROI2	-2.47E-03	3.21E-03	181	-7.68E-01	7.23E-01	
Year 0 - Year 2	Control	ROI2	-1.98E-03	3.21E-03	181	-6.15E-01	8.12E-01	
Year 1 - Year 2	Control	ROI2	4.89E-04	3.21E-03	181	1.52E-01	9.87E-01	
Year 0 - Year 1	Fibers	ROI2	-3.47E-03	3.37E-03	196	-1.03E+00	5.60E-01	
Year 0 - Year 2	Fibers	ROI2	-2.14E-03	3.79E-03	223	-5.64E-01	8.39E-01	
Year 1 - Year 2	Fibers	ROI2	1.33E-03	3.75E-03	221	3.55E-01	9.33E-01	
Year 0 - Year 1	Fragments	ROI2	-2.40E-03	3.43E-03	201	-6.99E-01	7.64E-01	
Year 0 - Year 2	Fragments	ROI2	-6.70E-04	3.94E-03	234	-1.70E-01	9.84E-01	
Year 1 - Year 2	Fragments	ROI2	1.73E-03	3.93E-03	231	4.40E-01	8.99E-01	
Year 0 - Year 1	Control	ROI3	-1.66E-03	3.21E-03	181	-5.17E-01	8.63E-01	
Year 0 - Year 2	Control	ROI3	1.43E-02	3.21E-03	181	4.46E+00	4.31E-05	***
Year 1 - Year 2	Control	ROI3	1.60E-02	3.21E-03	181	4.97E+00	4.52E-06	***
Year 0 - Year 1	Fibers	ROI3	4.44E-04	3.31E-03	190	1.34E-01	9.90E-01	
Year 0 - Year 2	Fibers	ROI3	1.47E-02	3.89E-03	229	3.78E+00	5.84E-04	***
Year 1 - Year 2	Fibers	ROI3	1.42E-02	3.89E-03	229	3.67E+00	8.92E-04	***
Year 0 - Year 1	Fragments	ROI3	-2.65E-03	3.31E-03	190	-8.00E-01	7.03E-01	
Year 0 - Year 2	Fragments	ROI3	1.10E-02	3.88E-03	229	2.83E+00	1.41E-02	*
Year 1 - Year 2	Fragments	ROI3	1.36E-02	3.88E-03	229	3.51E+00	1.56E-03	**

Table S10.1: Summary of statistical analyses from a three-way ANOVA assessing the effects of replicate, ROI, year, and their interactions on mean distance of soil matrix to next aerated macropore connected to the top. The table includes information on sums of squares, mean squares, degrees of freedom, F-values, and p-values for each main effect and interaction. Statistical significance is indicated as follows: p-value <0.001 ***, <0.01 **, <0.05 *, and <0.1 •.

Mean Distance							
**ANOVA Results**							
	**Sum Sq**	**Mean Sq**	**NumDF**	**DenDF**	**F value**	**Pr(>F)**	**Significance**
Replicate	2.49E-01	1.24E-01	2	2.53E+02	1.61E+01	2.58E-07	***
ROI	9.13E-02	4.56E-02	2	3.02E+01	5.92E+00	6.80E-03	**
Year	1.30E+00	6.50E-01	2	2.43E+01	8.43E+01	1.17E-11	***
Replicate:ROI	1.21E-01	3.04E-02	4	2.45E+02	3.94E+00	4.08E-03	**
Replicate:Year	1.12E-01	2.81E-02	4	2.54E+02	3.64E+00	6.69E-03	**
ROI:Year	1.59E-01	3.98E-02	4	2.44E+02	5.16E+00	5.23E-04	***
Replicate:ROI:Year	1.54E-01	1.92E-02	8	2.40E+02	2.49E+00	1.29E-02	*
**Scaled Residuals**							
	**Min.**	**1st Qu.**	**Median**	**Mean**	**3rd Qu.**	**Max.**	
**Freq**	-2.50E+00	-6.19E-01	-1.18E-01	-6.61E-18	5.74E-01	4.60E+00	
**Random Effects**							
**grp**	**var1**	**vcov**	**sdcor**	**Std.Dev.**			
Block:ROI	(Intercept)	2.08E-04	1.44E-02	1.44E-02			
Block:Year	(Intercept)	1.22E-03	3.49E-02	3.49E-02			
Block	(Intercept)	9.34E-04	3.06E-02	3.06E-02			
Residual		7.72E-03	8.78E-02	8.78E-02			
**Fixed Effects**							
	**Estimate**	**Std. Error**	**df**	**t value**	**Pr(>|t|)**	**Significance**	
(Intercept)	6.04E-01	2.59E-02	206	2.33E+01	1.33E-59	***	
ReplicateFibers	-5.81E-02	3.27E-02	234	-1.78E+00	7.69E-02	.	
ReplicateFragments	-3.84E-02	3.42E-02	239	-1.12E+00	2.63E-01		
ROIROI2	9.49E-02	3.31E-02	251	2.86E+00	4.54E-03	**	
ROIROI3	-2.26E-02	3.31E-02	251	-6.81E-01	4.96E-01		
YearYear 1	9.39E-02	3.45E-02	201	2.72E+00	7.12E-03	**	
YearYear 2	2.66E-01	3.51E-02	206	7.56E+00	1.27E-12	***	
ReplicateFibers:ROIROI2	-1.66E-01	4.76E-02	235	-3.49E+00	5.68E-04	***	
ReplicateFragments:ROIROI2	-1.50E-01	4.88E-02	240	-3.07E+00	2.39E-03	**	
ReplicateFibers:ROIROI3	3.96E-02	4.66E-02	234	8.50E-01	3.96E-01		
ReplicateFragments:ROIROI3	5.22E-02	4.86E-02	238	1.07E+00	2.84E-01		
ReplicateFibers:YearYear 1	1.18E-01	4.62E-02	233	2.56E+00	1.11E-02	*	
ReplicateFragments:YearYear 1	1.05E-02	4.83E-02	236	2.17E-01	8.28E-01		
ReplicateFibers:YearYear 2	3.14E-04	5.16E-02	251	6.09E-03	9.95E-01		
ReplicateFragments:YearYear 2	-5.96E-02	5.13E-02	247	-1.16E+00	2.46E-01		
ROIROI2:YearYear 1	-4.42E-02	4.58E-02	233	-9.65E-01	3.36E-01		
ROIROI3:YearYear 1	1.23E-02	4.58E-02	232	2.69E-01	7.88E-01		
ROIROI2:YearYear 2	-1.95E-01	4.68E-02	238	-4.18E+00	4.16E-05	***	
ROIROI3:YearYear 2	7.09E-02	4.66E-02	234	1.52E+00	1.30E-01		
ReplicateFibers:ROIROI2:YearYear 1	3.22E-02	6.71E-02	233	4.80E-01	6.32E-01		
ReplicateFragments:ROIROI2:YearYear 1	1.05E-01	6.79E-02	235	1.55E+00	1.22E-01		
ReplicateFibers:ROIROI3:YearYear 1	-8.78E-02	6.81E-02	237	-1.29E+00	1.99E-01		
ReplicateFragments:ROIROI3:YearYear 1	-3.03E-02	6.88E-02	233	-4.40E-01	6.60E-01		
ReplicateFibers:ROIROI2:YearYear 2	1.72E-01	7.31E-02	244	2.35E+00	1.94E-02	*	
ReplicateFragments:ROIROI2:YearYear 2	2.09E-01	7.24E-02	245	2.88E+00	4.30E-03	**	
ReplicateFibers:ROIROI3:YearYear 2	-3.12E-02	7.23E-02	241	-4.32E-01	6.66E-01		
ReplicateFragments:ROIROI3:YearYear 2	-7.04E-02	7.21E-02	241	-9.76E-01	3.30E-01		

Table S10.2: Post-hoc comparisons for mean distance of soil matrix to next aerated macropore connected to the top showing pairwise differences across replicate, ROI, and years. Compairing between replicates within each ROI and year, and temporal differences across years within each replicate and ROI. Results include estimates, standard errors (SE), degrees of freedom (df), t-ratios, p-values, and significance levels, highlighting significant differences over time and between groups. Statistical significance is indicated as follows: p-value <0.001 ***, <0.01 **, <0.05 *, and <0.1 •.

Mean Distance								
Post-hoc Comparisons								
Contrast	ROI	Year	Estimate	SE	df	t.ratio	p.value	Significance
Control - Fibers	ROI1	Year 0	5.81E-02	3.27E-02	236	1.78E+00	1.80E-01	
Control - Fragments	ROI1	Year 0	3.84E-02	3.43E-02	241	1.12E+00	5.02E-01	
Fibers - Fragments	ROI1	Year 0	-1.97E-02	3.49E-02	244	-5.64E-01	8.40E-01	
Control - Fibers	ROI2	Year 0	2.24E-01	3.47E-02	239	6.46E+00	1.71E-09	***
Control - Fragments	ROI2	Year 0	1.88E-01	3.49E-02	244	5.39E+00	4.93E-07	***
Fibers - Fragments	ROI2	Year 0	-3.63E-02	3.65E-02	251	-9.96E-01	5.80E-01	
Control - Fibers	ROI3	Year 0	1.85E-02	3.34E-02	238	5.55E-01	8.44E-01	
Control - Fragments	ROI3	Year 0	-1.37E-02	3.49E-02	244	-3.94E-01	9.18E-01	
Fibers - Fragments	ROI3	Year 0	-3.22E-02	3.49E-02	244	-9.23E-01	6.26E-01	
Control - Fibers	ROI1	Year 1	-6.03E-02	3.27E-02	236	-1.84E+00	1.58E-01	
Control - Fragments	ROI1	Year 1	2.79E-02	3.43E-02	241	8.15E-01	6.94E-01	
Fibers - Fragments	ROI1	Year 1	8.82E-02	3.49E-02	244	2.53E+00	3.25E-02	*
Control - Fibers	ROI2	Year 1	7.39E-02	3.43E-02	241	2.16E+00	8.09E-02	.
Control - Fragments	ROI2	Year 1	7.23E-02	3.27E-02	236	2.21E+00	7.17E-02	.
Fibers - Fragments	ROI2	Year 1	-1.61E-03	3.49E-02	244	-4.60E-02	9.99E-01	
Control - Fibers	ROI3	Year 1	-1.21E-02	3.76E-02	250	-3.21E-01	9.45E-01	
Control - Fragments	ROI3	Year 1	6.03E-03	3.52E-02	244	1.71E-01	9.84E-01	
Fibers - Fragments	ROI3	Year 1	1.81E-02	4.05E-02	259	4.47E-01	8.96E-01	
Control - Fibers	ROI1	Year 2	5.78E-02	4.01E-02	264	1.44E+00	3.22E-01	
Control - Fragments	ROI1	Year 2	9.80E-02	3.83E-02	253	2.56E+00	2.94E-02	*
Fibers - Fragments	ROI1	Year 2	4.02E-02	4.33E-02	247	9.30E-01	6.22E-01	
Control - Fibers	ROI2	Year 2	5.21E-02	4.05E-02	272	1.29E+00	4.04E-01	
Control - Fragments	ROI2	Year 2	3.92E-02	3.89E-02	269	1.01E+00	5.72E-01	
Fibers - Fragments	ROI2	Year 2	-1.29E-02	4.32E-02	246	-2.98E-01	9.52E-01	
Control - Fibers	ROI3	Year 2	4.94E-02	4.01E-02	265	1.23E+00	4.36E-01	
Control - Fragments	ROI3	Year 2	1.16E-01	3.83E-02	254	3.04E+00	7.38E-03	**
Fibers - Fragments	ROI3	Year 2	6.69E-02	4.33E-02	248	1.55E+00	2.72E-01	
Contrast	Replicate	ROI	Estimate	SE	df	t.ratio	p.value	Significance
Year 0 - Year 1	Control	ROI1	-9.39E-02	3.45E-02	208	-2.72E+00	1.94E-02	*
Year 0 - Year 2	Control	ROI1	-2.66E-01	3.51E-02	212	-7.56E+00	3.58E-12	***
Year 1 - Year 2	Control	ROI1	-1.72E-01	3.51E-02	212	-4.89E+00	5.95E-06	***
Year 0 - Year 1	Fibers	ROI1	-2.12E-01	3.57E-02	214	-5.95E+00	3.22E-08	***
Year 0 - Year 2	Fibers	ROI1	-2.66E-01	4.19E-02	245	-6.35E+00	3.14E-09	***
Year 1 - Year 2	Fibers	ROI1	-5.37E-02	4.19E-02	245	-1.28E+00	4.07E-01	
Year 0 - Year 1	Fragments	ROI1	-1.04E-01	3.84E-02	227	-2.72E+00	1.92E-02	*
Year 0 - Year 2	Fragments	ROI1	-2.06E-01	4.17E-02	247	-4.94E+00	4.32E-06	***
Year 1 - Year 2	Fragments	ROI1	-1.02E-01	4.17E-02	247	-2.44E+00	4.09E-02	*
Year 0 - Year 1	Control	ROI2	-4.97E-02	3.51E-02	213	-1.41E+00	3.35E-01	
Year 0 - Year 2	Control	ROI2	-7.02E-02	3.59E-02	218	-1.96E+00	1.26E-01	
Year 1 - Year 2	Control	ROI2	-2.05E-02	3.53E-02	213	-5.81E-01	8.30E-01	
Year 0 - Year 1	Fibers	ROI2	-2.00E-01	3.85E-02	234	-5.20E+00	1.27E-06	***
Year 0 - Year 2	Fibers	ROI2	-2.43E-01	4.31E-02	249	-5.63E+00	1.47E-07	***
Year 1 - Year 2	Fibers	ROI2	-4.23E-02	4.30E-02	246	-9.83E-01	5.88E-01	
Year 0 - Year 1	Fragments	ROI2	-1.66E-01	3.71E-02	224	-4.46E+00	3.79E-05	***
Year 0 - Year 2	Fragments	ROI2	-2.19E-01	4.16E-02	243	-5.26E+00	9.31E-07	***
Year 1 - Year 2	Fragments	ROI2	-5.36E-02	4.04E-02	238	-1.33E+00	3.82E-01	
Year 0 - Year 1	Control	ROI3	-1.06E-01	3.51E-02	213	-3.02E+00	7.86E-03	**
Year 0 - Year 2	Control	ROI3	-3.37E-01	3.57E-02	217	-9.42E+00	0.00E+00	***
Year 1 - Year 2	Control	ROI3	-2.30E-01	3.51E-02	212	-6.56E+00	1.23E-09	***
Year 0 - Year 1	Fibers	ROI3	-1.37E-01	4.02E-02	243	-3.40E+00	2.26E-03	**
Year 0 - Year 2	Fibers	ROI3	-3.06E-01	4.19E-02	245	-7.30E+00	1.23E-11	***
Year 1 - Year 2	Fibers	ROI3	-1.69E-01	4.60E-02	266	-3.67E+00	8.39E-04	***
Year 0 - Year 1	Fragments	ROI3	-8.64E-02	3.92E-02	232	-2.20E+00	7.27E-02	.
Year 0 - Year 2	Fragments	ROI3	-2.07E-01	4.17E-02	247	-4.95E+00	4.07E-06	***
Year 1 - Year 2	Fragments	ROI3	-1.20E-01	4.25E-02	248	-2.83E+00	1.39E-02	*

Table S11.1: Summary of statistical analyses from a three-way ANOVA assessing the effects of replicate, layer, year, and their interactions on vertical transport of MPs. The table includes information on sums of squares, mean squares, degrees of freedom, F-values, and p-values for each main effect and interaction. Statistical significance is indicated as follows: p-value <0.001 ***, <0.01 **, <0.05 *, and <0.1 •.

**MPs Transport**							
**ANOVA Results**							
	**Sum Sq**	**Mean Sq**	**NumDF**	**DenDF**	**F value**	**Pr(>F)**	**Significance**
Replicate	9.96E-01	9.96E-01	1	2.12E+01	9.72E+00	5.16E-03	**
Layer	2.63E+02	6.57E+01	4	8.66E+01	6.41E+02	2.01E-63	***
Year	2.08E+00	2.08E+00	1	2.12E+01	2.03E+01	1.89E-04	***
Replicate:Layer	7.44E-01	1.86E-01	4	8.66E+01	1.81E+00	1.33E-01	
Replicate:Year	1.12E-01	1.12E-01	1	2.12E+01	1.10E+00	3.07E-01	
Layer:Year	4.74E+00	1.19E+00	4	8.66E+01	1.16E+01	1.43E-07	***
Replicate:Layer:Year	3.65E+00	9.13E-01	4	8.66E+01	8.91E+00	4.47E-06	***
**Scaled Residuals**							
	**Min.**	**1st Qu.**	**Median**	**Mean**	**3rd Qu.**	**Max.**	
**Freq**	-3.85E+00	-5.92E-01	-4.70E-03	-7.93E-19	6.11E-01	3.38E+00	
**Random Effects**							
**grp**	**var1**	**vcov**	**sdcor**	**Std.Dev.**			
Block:Replicate	(Intercept)	4.13E-03	6.43E-02	6.43E-02			
Residual	NA	1.02E-01	3.20E-01	3.20E-01			
**Fixed Effects**							
	**Estimate**	**Std. Error**	**df**	**t value**	**Pr(>|t|)**	**Significance**	
(Intercept)	4.39E+00	1.46E-01	109	3.01E+01	1.03E-54	***	
ReplicateFragments	-1.80E-01	2.06E-01	109	-8.74E-01	3.84E-01		
Layer2	-1.93E+00	2.02E-01	84	-9.54E+00	4.95E-15	***	
Layer3	-3.92E+00	2.02E-01	84	-1.94E+01	1.15E-32	***	
Layer4	-4.11E+00	2.15E-01	88	-1.91E+01	4.67E-33	***	
Layer5	-4.13E+00	2.35E-01	93	-1.76E+01	2.73E-31	***	
YearYear 2	-6.50E-01	1.91E-01	109	-3.40E+00	9.40E-04	***	
ReplicateFragments:Layer2	1.20E+00	2.95E-01	86	4.05E+00	1.13E-04	***	
ReplicateFragments:Layer3	4.66E-01	2.86E-01	84	1.63E+00	1.07E-01		
ReplicateFragments:Layer4	2.47E-01	2.95E-01	86	8.35E-01	4.06E-01		
ReplicateFragments:Layer5	3.40E-01	3.10E-01	89	1.10E+00	2.75E-01		
ReplicateFragments:YearYear 2	3.72E-01	2.64E-01	109	1.41E+00	1.62E-01		
Layer2:YearYear 2	1.95E+00	2.65E-01	84	7.34E+00	1.25E-10	***	
Layer3:YearYear 2	1.00E+00	2.65E-01	86	3.77E+00	2.99E-04	***	
Layer4:YearYear 2	1.19E+00	2.69E-01	88	4.42E+00	2.82E-05	***	
Layer5:YearYear 2	9.11E-01	2.85E-01	91	3.20E+00	1.91E-03	**	
ReplicateFragments:Layer2:YearYear 2	-1.90E+00	3.73E-01	85	-5.09E+00	2.08E-06	***	
ReplicateFragments:Layer3:YearYear 2	1.15E-03	3.66E-01	85	3.13E-03	9.98E-01		
ReplicateFragments:Layer4:YearYear 2	-2.57E-01	3.69E-01	86	-6.97E-01	4.88E-01		
ReplicateFragments:Layer5:YearYear 2	-3.80E-01	3.81E-01	88	-9.98E-01	3.21E-01		

Table S11.2: Post-hoc comparisons for vertical transport of MPs showing pairwise differences across replicate, layer, and years. Compairing between fibers and fragments within each layer and year, and temporal differences across years within each replicate and layer. Results include estimates, standard errors (SE), degrees of freedom (df), t-ratios, p-values, and significance levels, highlighting significant differences over time and between groups. Statistical significance is indicated as follows: p-value <0.001 ***, <0.01 **, <0.05 *, and <0.1 •.

MPs Transport								
Post-hoc Comparisons								
Contrast	Year	Layer	Estimate	SE	df	t.ratio	p.value	Significance
Fibers - Fragments	Year 1	1	1.80E-01	2.06E-01	109	8.74E-01	3.84E-01	
Fibers - Fragments	Year 2	1	-1.92E-01	1.65E-01	110	-1.16E+00	2.47E-01	
Fibers - Fragments	Year 1	2	-1.02E+00	2.19E-01	110	-4.63E+00	1.01E-05	***
Fibers - Fragments	Year 2	2	5.14E-01	1.65E-01	110	3.12E+00	2.32E-03	**
Fibers - Fragments	Year 1	3	-2.86E-01	2.06E-01	109	-1.38E+00	1.69E-01	
Fibers - Fragments	Year 2	3	-6.59E-01	1.65E-01	110	-4.00E+00	1.15E-04	***
Fibers - Fragments	Year 1	4	-6.62E-02	2.19E-01	110	-3.02E-01	7.63E-01	
Fibers - Fragments	Year 2	4	-1.81E-01	1.54E-01	109	-1.18E+00	2.42E-01	
Fibers - Fragments	Year 1	5	-1.60E-01	2.39E-01	110	-6.69E-01	5.05E-01	
Fibers - Fragments	Year 2	5	-1.52E-01	1.54E-01	109	-9.87E-01	3.26E-01	
Contrast	Replicate	Layer	Estimate	SE	df	t.ratio	p.value	Significance
Year 1 - Year 2	Fibers	1	6.50E-01	1.91E-01	110	3.40E+00	9.52E-04	***
Year 1 - Year 2	Fragments	1	2.78E-01	1.82E-01	109	1.53E+00	1.30E-01	
Year 1 - Year 2	Fibers	2	-1.30E+00	1.91E-01	110	-6.77E+00	6.50E-10	***
Year 1 - Year 2	Fragments	2	2.33E-01	1.97E-01	110	1.18E+00	2.39E-01	
Year 1 - Year 2	Fibers	3	-3.50E-01	1.91E-01	110	-1.83E+00	6.99E-02	.
Year 1 - Year 2	Fragments	3	-7.23E-01	1.82E-01	109	-3.97E+00	1.27E-04	***
Year 1 - Year 2	Fibers	4	-5.39E-01	1.97E-01	110	-2.74E+00	7.12E-03	**
Year 1 - Year 2	Fragments	4	-6.54E-01	1.82E-01	109	-3.59E+00	4.97E-04	***
Year 1 - Year 2	Fibers	5	-2.61E-01	2.19E-01	110	-1.19E+00	2.35E-01	
Year 1 - Year 2	Fragments	5	-2.53E-01	1.82E-01	109	-1.39E+00	1.68E-01	

Table S11.3: Post-hoc comparisons for vertical transport of MPs showing pairwise differences across replicate, layer, and years. Compairing between layers within each replicate and year. Results include estimates, standard errors (SE), degrees of freedom (df), t-ratios, p-values, and significance levels, highlighting significant differences over time and between groups. Statistical significance is indicated as follows: p-value <0.001 ***, <0.01 **, <0.05 *, and <0.1 •.

MPs Transport								
Post-hoc Comparisons								
Contrast	Replicate	Year	Estimate	SE	df	t.ratio	p.value	Significance
Layer1 - Layer2	Fibers	Year 1	1.93E+00	2.02E-01	87	9.54E+00	2.89E-10	***
Layer1 - Layer3	Fibers	Year 1	3.92E+00	2.02E-01	87	1.94E+01	2.89E-10	***
Layer1 - Layer4	Fibers	Year 1	4.11E+00	2.15E-01	90	1.91E+01	4.48E-10	***
Layer1 - Layer5	Fibers	Year 1	4.13E+00	2.36E-01	95	1.75E+01	4.73E-10	***
Layer2 - Layer3	Fibers	Year 1	1.99E+00	2.02E-01	87	9.82E+00	2.89E-10	***
Layer2 - Layer4	Fibers	Year 1	2.18E+00	2.15E-01	90	1.01E+01	4.48E-10	***
Layer2 - Layer5	Fibers	Year 1	2.20E+00	2.36E-01	95	9.34E+00	4.73E-10	***
Layer3 - Layer4	Fibers	Year 1	1.92E-01	2.15E-01	90	8.92E-01	8.99E-01	
Layer3 - Layer5	Fibers	Year 1	2.13E-01	2.36E-01	95	9.05E-01	8.94E-01	
Layer4 - Layer5	Fibers	Year 1	2.10E-02	2.46E-01	91	8.54E-02	1.00E+00	
Layer1 - Layer2	Fragments	Year 1	7.36E-01	2.16E-01	90	3.41E+00	8.36E-03	**
Layer1 - Layer3	Fragments	Year 1	3.45E+00	2.02E-01	87	1.71E+01	2.89E-10	***
Layer1 - Layer4	Fragments	Year 1	3.86E+00	2.02E-01	87	1.91E+01	2.89E-10	***
Layer1 - Layer5	Fragments	Year 1	3.79E+00	2.02E-01	87	1.87E+01	2.89E-10	***
Layer2 - Layer3	Fragments	Year 1	2.72E+00	2.16E-01	90	1.26E+01	4.40E-10	***
Layer2 - Layer4	Fragments	Year 1	3.13E+00	2.16E-01	90	1.45E+01	4.40E-10	***
Layer2 - Layer5	Fragments	Year 1	3.06E+00	2.16E-01	90	1.42E+01	4.40E-10	***
Layer3 - Layer4	Fragments	Year 1	4.12E-01	2.02E-01	87	2.04E+00	2.58E-01	
Layer3 - Layer5	Fragments	Year 1	3.39E-01	2.02E-01	87	1.68E+00	4.55E-01	
Layer4 - Layer5	Fragments	Year 1	-7.29E-02	2.02E-01	87	-3.60E-01	9.96E-01	
Layer1 - Layer2	Fibers	Year 2	-1.47E-02	1.71E-01	87	-8.59E-02	1.00E+00	
Layer1 - Layer3	Fibers	Year 2	2.92E+00	1.72E-01	91	1.70E+01	4.72E-10	***
Layer1 - Layer4	Fibers	Year 2	2.92E+00	1.62E-01	91	1.80E+01	4.64E-10	***
Layer1 - Layer5	Fibers	Year 2	3.22E+00	1.62E-01	91	1.99E+01	4.64E-10	***
Layer2 - Layer3	Fibers	Year 2	2.93E+00	1.72E-01	91	1.71E+01	4.72E-10	***
Layer2 - Layer4	Fibers	Year 2	2.94E+00	1.62E-01	91	1.81E+01	4.64E-10	***
Layer2 - Layer5	Fibers	Year 2	3.24E+00	1.62E-01	91	2.00E+01	4.64E-10	***
Layer3 - Layer4	Fibers	Year 2	3.62E-03	1.62E-01	91	2.24E-02	1.00E+00	
Layer3 - Layer5	Fibers	Year 2	3.02E-01	1.62E-01	91	1.87E+00	3.42E-01	
Layer4 - Layer5	Fibers	Year 2	2.99E-01	1.51E-01	87	1.98E+00	2.84E-01	
Layer1 - Layer2	Fragments	Year 2	6.91E-01	1.51E-01	87	4.58E+00	1.50E-04	***
Layer1 - Layer3	Fragments	Year 2	2.45E+00	1.51E-01	87	1.62E+01	2.89E-10	***
Layer1 - Layer4	Fragments	Year 2	2.93E+00	1.51E-01	87	1.94E+01	2.89E-10	***
Layer1 - Layer5	Fragments	Year 2	3.26E+00	1.51E-01	87	2.16E+01	2.89E-10	***
Layer2 - Layer3	Fragments	Year 2	1.76E+00	1.51E-01	87	1.17E+01	2.89E-10	***
Layer2 - Layer4	Fragments	Year 2	2.24E+00	1.51E-01	87	1.49E+01	2.89E-10	***
Layer2 - Layer5	Fragments	Year 2	2.57E+00	1.51E-01	87	1.70E+01	2.89E-10	***
Layer3 - Layer4	Fragments	Year 2	4.82E-01	1.51E-01	87	3.19E+00	1.64E-02	*
Layer3 - Layer5	Fragments	Year 2	8.10E-01	1.51E-01	87	5.37E+00	6.51E-06	***
Layer4 - Layer5	Fragments	Year 2	3.28E-01	1.51E-01	87	2.17E+00	2.00E-01	

Table S12.1: Summary of statistical analyses from a three-way ANOVA assessing the effects of crop type (Material), replicate, and their interactions on total biomass. The table includes information on sums of squares, mean squares, degrees of freedom, F-values, and p-values for each main effect and interaction. Statistical significance is indicated as follows: p-value <0.001 ***, <0.01 **, <0.05 *, and <0.1 •.

**Total Biomass**							
**ANOVA Results**							
	**Sum Sq**	**Mean Sq**	**NumDF**	**DenDF**	**F value**	**Pr(>F)**	**Significance**
Replicate	4.72E+01	2.36E+01	2	5.40E+01	3.37E-01	7.15E-01	
Material	8.41E+02	8.41E+02	1	5.59E+01	1.20E+01	1.01E-03	**
Replicate:Material	8.26E+02	4.13E+02	2	5.25E+01	5.91E+00	4.85E-03	**
**Scaled Residuals**							
	**Min.**	**1st Qu.**	**Median**	**Mean**	**3rd Qu.**	**Max.**	
**Freq**	-1.96E+00	-7.67E-01	-6.05E-02	3.74E-17	7.34E-01	2.74E+00	
**Random Effects**							
**grp**	**var1**	**vcov**	**sdcor**	**Std.Dev.**			
Block:Material	(Intercept)	0.00E+00	0.00E+00	0.00E+00			
Block	(Intercept)	6.02E+00	2.45E+00	2.45E+00			
Residual	NA	6.99E+01	8.36E+00	8.36E+00			
**Fixed Effects**							
	**Estimate**	**Std. Error**	**df**	**t value**	**Pr(>|t|)**	**Significance**	
(Intercept)	2.03E+01	2.33E+00	63	8.71E+00	2.15E-12	***	
ReplicateFibers	-1.02E+01	4.13E+00	56	-2.47E+00	1.66E-02	*	
ReplicateFragments	-3.16E+00	3.60E+00	53	-8.79E-01	3.83E-01		
MaterialWheat	6.99E-01	3.17E+00	51	2.21E-01	8.26E-01		
ReplicateFibers:MaterialWheat	1.74E+01	5.24E+00	54	3.32E+00	1.60E-03	**	
ReplicateFragments:MaterialWheat	2.63E+00	4.79E+00	52	5.49E-01	5.85E-01		
**Post-hoc Comparisons**							
**contrast**	**Material**	**Estimate**	**SE**	**df**	**t.ratio**	**p.value**	**Significance**
Control - Fibers	Barley	1.02E+01	4.24E+00	51	2.41E+00	5.10E-02	.
Control - Fragments	Barley	3.16E+00	3.65E+00	46	8.67E-01	6.64E-01	
Fibers - Fragments	Barley	-7.04E+00	4.55E+00	49	-1.55E+00	2.78E-01	
Control - Fibers	Wheat	-7.22E+00	3.25E+00	42	-2.22E+00	7.95E-02	.
Control - Fragments	Wheat	5.30E-01	3.18E+00	41	1.67E-01	9.85E-01	
Fibers - Fragments	Wheat	7.75E+00	3.25E+00	42	2.38E+00	5.57E-02	.

Table S12.2: Summary of statistical analyses from a three-way ANOVA assessing the effects of crop type (Material), replicate, and their interactions on grain weight. The table includes information on sums of squares, mean squares, degrees of freedom, F-values, and p-values for each main effect and interaction. Statistical significance is indicated as follows: p-value <0.001 ***, <0.01 **, <0.05 *, and <0.1 •.

Grain Weight							
**ANOVA Results**							
	**Sum Sq**	**Mean Sq**	**NumDF**	**DenDF**	**F value**	**Pr(>F)**	**Significance**
Replicate	1.26E+01	6.30E+00	2	5.42E+01	4.20E-01	6.59E-01	
Material	3.45E+01	3.45E+01	1	5.76E+01	2.30E+00	1.35E-01	
Replicate:Material	1.43E+02	7.16E+01	2	5.38E+01	4.77E+00	1.23E-02	*
**Scaled Residuals**							
	**Min.**	**1st Qu.**	**Median**	**Mean**	**3rd Qu.**	**Max.**	
**Freq**	-2.17E+00	-7.20E-01	-6.32E-02	2.06E-17	7.77E-01	2.24E+00	
**Random Effects**							
**grp**	**var1**	**vcov**	**sdcor**	**Std.Dev.**			
Block:Material	(Intercept)	0.00E+00	0.00E+00	0.00E+00			
Block	(Intercept)	1.08E+00	1.04E+00	1.04E+00			
Residual	NA	1.50E+01	3.87E+00	3.87E+00			
**Fixed Effects**							
	**Estimate**	**Std. Error**	**df**	**t value**	**Pr(>|t|)**	**Significance**	
(Intercept)	1.02E+01	1.07E+00	64	9.56E+00	5.93E-14	***	
ReplicateFibers	-4.28E+00	1.81E+00	56	-2.37E+00	2.14E-02	*	
ReplicateFragments	-1.69E+00	1.67E+00	55	-1.02E+00	3.14E-01		
MaterialWheat	-1.39E+00	1.47E+00	52	-9.47E-01	3.48E-01		
ReplicateFibers:MaterialWheat	7.09E+00	2.35E+00	55	3.02E+00	3.85E-03	**	
ReplicateFragments:MaterialWheat	1.46E+00	2.22E+00	54	6.59E-01	5.13E-01		
**Post-hoc Comparisons**							
**contrast**	**Material**	**Estimate**	**SE**	**df**	**t.ratio**	**p.value**	**Significance**
Control - Fibers	Barley	4.28E+00	1.85E+00	50	2.32E+00	6.25E-02	.
Control - Fragments	Barley	1.69E+00	1.69E+00	47	1.00E+00	5.78E-01	
Fibers - Fragments	Barley	-2.59E+00	2.00E+00	47	-1.30E+00	4.04E-01	
Control - Fibers	Wheat	-2.81E+00	1.51E+00	43	-1.86E+00	1.61E-01	
Control - Fragments	Wheat	2.30E-01	1.47E+00	42	1.56E-01	9.87E-01	
Fibers - Fragments	Wheat	3.04E+00	1.51E+00	43	2.02E+00	1.20E-01	

Table S12.3: Summary of statistical analyses from a three-way ANOVA assessing the effects of crop type (Material), replicate, and their interactions on harvest index. The table includes information on sums of squares, mean squares, degrees of freedom, F-values, and p-values for each main effect and interaction. Statistical significance is indicated as follows: p-value <0.001 ***, <0.01 **, <0.05 *, and <0.1 •.

Harvest Index							
**ANOVA Results**							
	**Sum Sq**	**Mean Sq**	**NumDF**	**DenDF**	**F value**	**Pr(>F)**	**Significance**
Replicate	1.95E-03	9.77E-04	2	5.80E+01	1.25E+00	2.94E-01	
Material	1.00E-01	1.00E-01	1	5.80E+01	1.29E+02	2.39E-16	***
Replicate:Material	2.24E-03	1.12E-03	2	5.80E+01	1.43E+00	2.46E-01	
**Scaled Residuals**							
	**Min.**	**1st Qu.**	**Median**	**Mean**	**3rd Qu.**	**Max.**	
**Freq**	-4.19E+00	-4.47E-01	-1.89E-02	-2.23E-17	6.30E-01	2.54E+00	
**Random Effects**							
**grp**	**var1**	**vcov**	**sdcor**	**Std.Dev.**			
Block:Material	(Intercept)	0.00E+00	0.00E+00	0.00E+00			
Block	(Intercept)	0.00E+00	0.00E+00	0.00E+00			
Residual	NA	7.80E-04	2.79E-02	2.79E-02			
**Fixed Effects**							
	**Estimate**	**Std. Error**	**df**	**t value**	**Pr(>|t|)**	**Significance**	
(Intercept)	5.22E-01	8.06E-03	58	6.47E+01	8.96E-56	***	
ReplicateFibers	-2.63E-02	1.40E-02	58	-1.89E+00	6.44E-02	.	
ReplicateFragments	-2.13E-02	1.27E-02	58	-1.67E+00	1.01E-01		
MaterialWheat	-9.91E-02	1.12E-02	58	-8.86E+00	2.26E-12	***	
ReplicateFibers:MaterialWheat	2.83E-02	1.81E-02	58	1.57E+00	1.23E-01		
ReplicateFragments:MaterialWheat	2.07E-02	1.67E-02	58	1.24E+00	2.20E-01		
**Post-hoc Comparisons**							
**contrast**	**Material**	**Estimate**	**SE**	**df**	**t.ratio**	**p.value**	**Significance**
Control - Fibers	Barley	2.63E-02	1.46E-02	54	1.81E+00	1.77E-01	
Control - Fragments	Barley	2.13E-02	1.31E-02	46	1.62E+00	2.47E-01	
Fibers - Fragments	Barley	-5.07E-03	1.55E-02	47	-3.26E-01	9.43E-01	
Control - Fibers	Wheat	-1.93E-03	1.16E-02	38	-1.67E-01	9.85E-01	
Control - Fragments	Wheat	5.83E-04	1.08E-02	37	5.39E-02	9.98E-01	
Fibers - Fragments	Wheat	2.51E-03	1.14E-02	40	2.20E-01	9.74E-01	
